# Eicosapentaenoic acid influences the pathogenesis of *Candida* *albicans* in *Caenorhabditis* *elegans* via inhibition of hyphal formation and stimulation of the host immune response

**DOI:** 10.1007/s00430-023-00777-6

**Published:** 2023-09-06

**Authors:** N. Z. Mokoena, H. Steyn, A. Hugo, T. Dix-Peek, C. Dickens, O. M. N. Gcilitshana, O. Sebolai, J. Albertyn, C. H. Pohl

**Affiliations:** 1 Department of Microbiology and Biochemistry, University of the Free State, Bloemfontein, South Africa; 2 Department of Animal Science, University of the Free State, Bloemfontein, South Africa; 3 Department of Internal Medicine, University of Witwatersrand, Johannesburg, South Africa; 4 Department of Biochemistry, Genetics and Microbiology, University of Pretoria, Pretoria, South Africa

**Keywords:** Eicosapentaenoic acid, 17,18-Epoxyeicosatetraenoic acid, *Caenorhabditiselegans*, *Candidaalbicans*, Hyphal formation

## Abstract

The intake of omega-3 polyunsaturated fatty acids, including eicosapentaenoic acid (EPA), is associated with health benefits due to its anti-inflammatory properties. This fatty acid also exhibits antifungal properties in vitro. In order to determine if this antifungal property is valid in vivo*,* we examined how EPA affects *Candida* *albicans* pathogenesis in the *Caenorhabditis* *elegans* infection model, an alternative to mammalian host models. The nematodes were supplemented with EPA prior to infection, and the influence of EPA on *C.* *elegans* lipid metabolism, survival and immune response was studied. In addition, the influence of EPA on hyphal formation in *C.* *albicans* was investigated. It was discovered that EPA supplementation changed the lipid composition, but not the unsaturation index of *C.* *elegans* by regulating genes involved in fatty acid and eicosanoid production. EPA supplementation also delayed killing of *C.* *elegans* by *C.* *albicans* due to the inhibition of hyphal formation in vivo, via the action of the eicosanoid metabolite of EPA, 17,18-epoxyeicosatetraenoic acid. Moreover, EPA supplementation also caused differential expression of biofilm-related gene expression in *C.* *albicans* and stimulated the immune response of *C.* *elegans*. This provides a link between EPA and host susceptibility to microbial infection in this model.

**Supplementary Information:**

The online version contains supplementary material available at 10.1007/s00430-023-00777-6.

## Introduction

One of the central challenges found worldwide is finding ways to overcome the rapid increasing emergence of drug-resistant microorganisms. A diverse group of fatty acids are known to possess a protective role in nature as antimicrobial agents and ecological modulators that control microbial biofilms and virulence [1]. Polyunsaturated fatty acids (PUFAs), including omega-3 (n-3) and omega-6 (n-6) fatty acids, possess pleiotropic effects with numerous metabolic benefits, including the improvement of the immune system, as well as cardiovascular and neurologic health in mammals [2]. Numerous studies have documented the protective role of the n-3 PUFAs, eicosapentaenoic acid (EPA, 20:5n-3) and docosahexaenoic acid (22:6n-3), against cardiovascular and inflammatory/autoimmune diseases [3–6]. In addition to these benefits observed in disease control and prevention, to date, most of the natural PUFAs are becoming increasingly relevant as antimicrobial agents [7]. For instance, there have been reports on the antimicrobial activities of linoleic acid (18:2n-6), arachidonic acid (20:4n-6), EPA and 22:6n-3 against various pathogenic bacteria, including *Neisseria* *gonorrhoeae* [8], *Staphylococcus* *aureus* [9], *Pseudomonas* *aeruginosa* [10], *Burkholderia* *cenocepacia* [11], and *Helicobacter* *pylori* [12].

Recent studies indicate that fatty acids also have potential as antibiofilm agents, as many fatty acids have been identified to either disrupt or inhibit the formation of biofilms by various pathogenic microorganisms, including *Candida* *albicans* [13–16], *Candida* *krusei* [17], *S.* *aureus* [18, 19], *Serratia* *marcescens* [20] and *Vibrio* spp. [21]. Moreover, mouse model in vivo studies demonstrated that oral supplementation with n-3 fatty acids is effective at controlling *P.* *aeruginosa* infections [10, 22]. Although these studies suggest a potential role for long-chain PUFAs in pathogenesis, the influence of these PUFAs on *C*. *albicans* infections remains understudied. Therefore, we sought to determine the influence of EPA on the pathogenesis of *C*. *albicans* in the invertebrate model, *Caenorhabditis* *elegans* by investigating its effect on *C.* *albicans* hyphal formation as well as *C.* *elegans* immune response.

Since the establishment of *C.* *elegans* model system, research towards usage of this infection model in the field of immunology, genetics, and host–pathogen interactions has expanded rapidly. The use of this model in studying fungal infections is well established as is also the case for *C.* *albicans* infections (reviewed in [23]). The *C.* *elegans* infection model has numerous technical advantages compared to mammalian infection models, including simple growth conditions, short reproductive cycle, self-fertile (hermaphrodite reproduces about 300 progeny), small brood sizes, ease of handling, relative low cost, simplicity of equipment, and no ethical considerations [24, 25]. In addition, the nematode is transparent, and the simple and streamlined body structure offers a simplified in vivo model system that is able to mimic pathogenic and physiological mechanisms occurring during the time of infection, moreover, it also enables microscopic visualisation of internal events.

## Materials and methods

### Strains used

*Caenorhabditis* *elegans* *glp-4*; *sek-1* hermaphrodites, obtained from the *Caenorhabditis* Genetic Centre, College of Biological Sciences, University of Minnesota, were propagated on Nematode Growth Medium (NGM) (2.5 g/L peptone, 3 g/L sodium chloride, 17 g/L agar) spotted with *Escherichia* *coli* OP50 as food source [24]. The rationale behind the use of *C.* *elegans* *glp-4*; *sek-1* mutant in this study is that *glp-4* mutants cannot produce gonads or progeny at 25 °C, thereby preventing matricidal killing (hatching of eggs inside the nematode, leading to death of the nematode) at the assay temperature [26]. While *sek-1* mutants lack the gene that encodes a conserved mitogen-activated protein kinase involved in the innate immune response [27], thus making the mutant nematodes immunocompromised and readily infected with various pathogens. Since *glp-4* mutants are less susceptible to pathogens that wild-type nematodes, the additional mutation in *sek-1* provides a nematode with similar susceptibility to *C.* *albicans* than the wild type [25]. In all experiments, *E.* *coli* OP50 served as control. *Candida* *albicans* SC5314 was maintained on yeast extract-peptone-dextrose (YPD) agar (5 g/L peptone, 3 g/L yeast extract, 10 g/L glucose, 16 g/L agar) at 30 °C.

### Fatty acid supplementation of *C. elegans*

Nematode Growth Medium (NGM) agar was prepared with the addition of 10 ml of 0.1% Tergitol, which allows for even distribution of fatty acids through the entire plate and more efficient uptake of the fatty acids by *E.* *coli* and the nematodes [28]. Agar was cooled to 45–50 °C and 0.01 mM of EPA sodium salt was added slowly. Since the EPA sodium salt is water soluble, a solvent control was not included. Plates were poured immediately, covered to dry in the dark for 24 h, thereafter seeded with *E.* *coli* OP50, and incubated for 24 h in the dark at room temperature. Synchronised larva 1 (L1) *C.* *elegans* larvae that were grown for 4 days (in order to reach L4 stage) on NGM agar plates with or without 0.01 mM EPA, seeded with *E.* *coli* OP50, were carefully harvested and washed three times with sterile M9 buffer (6 g/L Na_2_HPO_4_, 3 g/L KH_2_PO_4_, 5 g/L NaCl, 0.25 g/L MgSO_4_.7H_2_O). Subsequently, the nematodes were transferred into sterile petri-dishes containing 10 ml liquid medium (80% M9 buffer, 20% Brain Heart Infusion [(BHI) (7.8 g/L brain extract, 9.7 g/L heart extract, 2.5 g/L disodium phosphate, 2.0 g/L dextrose)] and 90 μg/ml kanamycin) and incubated at 25 °C for 24 h.

### Fatty acid extraction and analyses

Following incubation, nematodes were washed by centrifugation at 4000*g* for 2 min, the supernatant gently aspirated, and the pellet containing the nematodes pulverised with a mortar and pestle to break open the nematodes for the release of their intracellular components. The total lipids of the nematodes were extracted overnight using chloroform/methanol (2:1 v/v). Thereafter, the extract was filtered, and the solvent phase, containing the lipids was removed, dried under nitrogen and stored at − 80 °C prior to analysis using gas chromatography. Fatty acids were transesterified to form methyl esters (FAMEs) using 0.5 N NaOH and 14% boron trifluoride in methanol [29]. Fatty acid methyl esters were quantified using a Varian 430 flame ionisation gas chromatography, with a fused silica capillary column, Chrompack CPSIL 88 (100 m length, 0.25 mm ID, 0.2 μm film thicknesses). All the analyses were performed using an initial isothermic period (40 °C for 2 min). Thereafter, the temperature was increased at a rate of 4 °C/min to 230 °C. This was followed by an isothermic period of 230 °C for 10 min. The FAMEs were then dissolved in *n*-hexane then 1 μl injected into the column using a Varian CP 8400 Autosampler. The injection port and detector were both maintained at a constant temperature of 250 °C. The hydrogen, at 45 psi, served as the carrier gas, while nitrogen served as the make-up gas. Finally, the chromatograms were recorded using the Galaxy Chromatography Software. The FAME samples were identified by comparing the retention times of authentic standards (Supelco 37 Component Fame Mix 47885-U, Sigma-Aldrich). The unsaturation indices of the extracted lipids of the control and supplemented was calculated as follows: Unsaturation Index = 1 × [% monoenoic fatty acids] + 2 × [% dienoic fatty acids] + 3 × [% trienoic fatty acids] + 4 × [% tetraenoic fatty acids] + 5 × [% pentaenoic fatty acids] [15].

### Infection of *C. elegans*

Infection was performed according to the protocol by Breger and co-workers [25]. Approximately 500 washed, synchronised L4 *C.* *elegans* nematodes, grown on NGM agar plates with or without 0.01 mM EPA, seeded with *E.* *coli* OP50, were placed on the centre of a *C.* *albicans* lawn grown on BHI agar, and incubated at 25 °C for 4 h. After incubation, nematodes were carefully transferred to conical tubes with sterile M9 buffer and washed three times with M9 buffer. Any microbial contaminants, which may confound the infection process, were removed via sucrose floatation during the washing step [30].

### *Caenorhabditis elegans* survival assay

After incubation, nematodes were harvested and washed as indicated above. Sixty nematodes were transferred into 2 ml liquid medium (80% M9 buffer, 20% BHI, 90 μg/ml kanamycin) in a single well of a six-well tissue culture plate. Nematodes were monitored daily by scoring them as either alive, dead or dead with hyphal formation. If nematodes did not show any movement in response to mechanical stimulation, they were considered dead and thus removed from liquid medium assay [25].

### Total RNA extraction

Nematodes were supplemented with EPA and infected with *C.* *albicans* as described above. Following this, nematodes were washed by centrifugation at 4000*g* for 2 min, the supernatant gently aspirated and 2 ml RNA*later* (Invitrogen) added to each sample to combat degradation of RNA. Samples were frozen at − 80 °C until RNA extraction. Samples were thawed on ice, centrifuged at 4000*g* for 2 min to collect cells and RNA*later* was aspirated. The nematode pellet was resuspended in 600 µl of lysis buffer (Zymo Research), supplemented with 1 volume of glass beads (diameter 0.5 mm) and mechanically homogenised twice for 15 min using a Disruptor Genie Analog Cell Disruptor. Total RNA was extracted from samples using Quick-RNA MiniPrep kit (Zymo Research), including removal of the genomic DNA by DNase digestion, according to manufacturer’s instructions. The RNA samples were evaluated using the Thermo Scientific NanoDrop ND-1000 Ultraviolet Visible Spectrophotometer to determine total RNA concentration in each sample.

### Analyses of differential expression with nCounter^®^

Total RNA extracted from the different treatment conditions were analysed with the NanoString nCounter^®^ analysis system [31], using a gene expression TagSet that targets 123 *C.* *albicans* genes—including three housekeeping genes, *ACT1*, *LSC2* and *THD3* [32]—and 60 *C.* *elegans* genes—including three housekeeping genes, *rps-2*, *rps-4* and *rps-23* [33]. The full list of genes with functions of the 183-genes can be found in Supplementary Table S1. Analyses of differential expression was performed using nCounter^®^ with Elements™ XT Reagents according to manufacturer’s specifications. A multiplexed probe library (nCounter^®^ elements CodeSet) was designed with two sequence-specific probes for each gene of interest. Probes were mixed with 100 ng of purified total RNA and allowed to hybridise (18 h, 67 °C). Samples were loaded on an nCounter^®^ SPRINT^™^ Cartridge and processed with an nCounter^®^ SPRINT^™^ Profiler (NanoString Technologies, USA) to quantify the transcripts. The nCounter raw expression data file (RCC) obtained was uploaded into the nSolver Analysis Software 4.0 for review of quality control metrics. The data were grouped between the experiments and control, and their expression ratios determined.

### Influence of 17,18-epoxyeicosatetraenoic acid on germ tube formation

*Candida* *albicans* yeast cells were grown on YPD agar plates and incubated at 37 °C overnight. Nematode broth was prepared by growing *C.* *elegans* until L4 stage on *E.* *coli* OP50 seeded NGM agar plates. Thereafter nematodes from 10 to 15 plates were carefully harvested and washed three times with sterile M9 buffer. The nematode pellet was resuspended in 1.5 ml M9 buffer, supplemented with glass beads (diameter 0.5 mm) and mechanically homogenised twice for 15 min using a Disruptor Genie Analog Cell Disruptor. The suspension was filtered using sterile syringe filter with a 0.2 µm pore size (GVS Filter Technology) to obtain nematode broth. Triplicate sets of test tubes containing either 5 ml of foetal bovine serum or nematode broth were inoculated with 2 to 3 colonies of *C.* *albicans*. Cell densities were adjusted to final cell concentration of approximately l0^6^ cells/ml. The cell suspension was supplemented with 0.01 mM 17,18-EpETE (Cayman Chemicals) and incubated at 37 °C for 4 h. The cells were washed three times and suspended in 5 ml of phosphate-buffered saline (PBS, pH 6.8). All samples were transferred to an ice bath at the end of the incubation period prior to microscopic observation and quantification of the percentage cells with germ tubes [34]. In addition, a crystal violet germ tube assay was performed in foetal bovine serum supplemented with 0.01 mM 17,18-EpETE. Briefly, *C.* *albicans* was grown on yeast malt (YM) agar plates and single colonies inoculated into YPD broth and incubated at 30 °C overnight. Cells were harvested by centrifugation and washed 3 times with sterile PBS. The cells were inoculated into foetal bovine serum and standardised to an OD_600_ of 0.8. A volume of 100 µl was inoculated into each well of a 96-well plate and 17,18-EpETE (final concentration of 0.01 mM) added to each well. The plate was incubated for 2 h at 37 °C, whereafter it was washed and the crystal violet assay was performed as described previously [35]. For all these experiments, the control contained the same amount of ethanol (solvent control).

To test the effect of 17,18-EpETE on *C.* *albicans* hyphal formation in *C.* *elegans*, a method reported by Tampakakis and co-workers [36] with slight modifications, was used. Briefly, synchronised L3 *C.* *elegans* nematodes that were grown for 24 h on *E.* *coli* OP50 seeded NGM agar plates with 17,18-EpETE added directly to the bacterial food at a final concentration of 0.01 mM. The control contained the same amount of ethanol (solvent control). Plates were then incubated for 24 h for nematodes to reach L4 stage. Thereafter, nematodes were carefully harvested and washed three times with sterile M9 buffer and infected with *C.* *albicans* [25]. Sixty nematodes were transferred into 2 ml of liquid medium (80% M9 buffer, 20% BHI, 90 μg/ml kanamycin) in a single well of a six-well tissue culture plate and incubated at 25 °C. Nematodes were monitored daily by scoring them as either alive or dead. If nematodes did not show any movement in response to mechanical stimulation, they were considered dead and thus removed from liquid medium assay [25]. Hyphal formation was monitored after 24 h of incubation.

### Influence of CYP inhibitors on hyphal formation in vivo

Nematode growth medium agar with 10 ml 0.1% Tergitol was supplemented with 0.01 mM of EPA as described. Synchronised L2 *C.* *elegans* nematodes were grown for 24 h on NGM agar plates with or without 0.01 mM EPA, seeded with *E.* *coli* OP50 until they reached L3 stage. Thereafter, the L3 nematodes were carefully harvested and washed three times with sterile M9 buffer. L3 nematodes were transferred to fresh plates and pre-treated for 24 h with 17-octadecynoic acid (17-ODYA) or 6-(2-propargyloxyphenyl)hexanoic acid (PPOH) (Cayman Chemicals) until they reached L4 stage. The compounds were added directly to the bacterial food at a final concentration of 0.05 mM [37, 38]. In all cases, solvent controls were included. L4 nematodes were infected with *C.* *albicans* and 60 nematodes were transferred into 2 ml of liquid medium (80% M9 buffer, 20% BHI, 90 μg/ml kanamycin) in a single well of a six-well tissue culture plate and incubated at 25 °C. Hyphal formation was monitored after 24 h of incubation. In addition, nematodes exposed to EPA and 17-ODYA were monitored for survival by scoring them as either alive or dead. If nematodes did not show any movement in response to mechanical stimulation, they were considered dead and thus removed from liquid medium assay [25].

### Statistical analysis

All experiments were executed in biological triplicates, each with three technical replicates. The averages and standard deviations were calculated. Student’s *t* test (two-tailed, unequal variance) was used to analyse the significance of differences between experimental groups. Data with a *P* value of ≤ 0.05 were considered to be significant. The *C*. *elegans* survival was assessed using the Kaplan–Meier method and differences were determined with the log-rank test using OASIS 2 with statistical analyses performed using two-way ANOVA with Bonferroni correction [39]. For NanoString nCounter^®^ analysis, biological triplicates were analysed and genes with a fold change of ≥ 1.5 or ≤ − 1.5 and *P* values ≤ 0.05 indicate a significant difference from control.

## Results 

### EPA supplementation influences nematode polyunsaturated fatty acid metabolism 

**Fig. 1:**
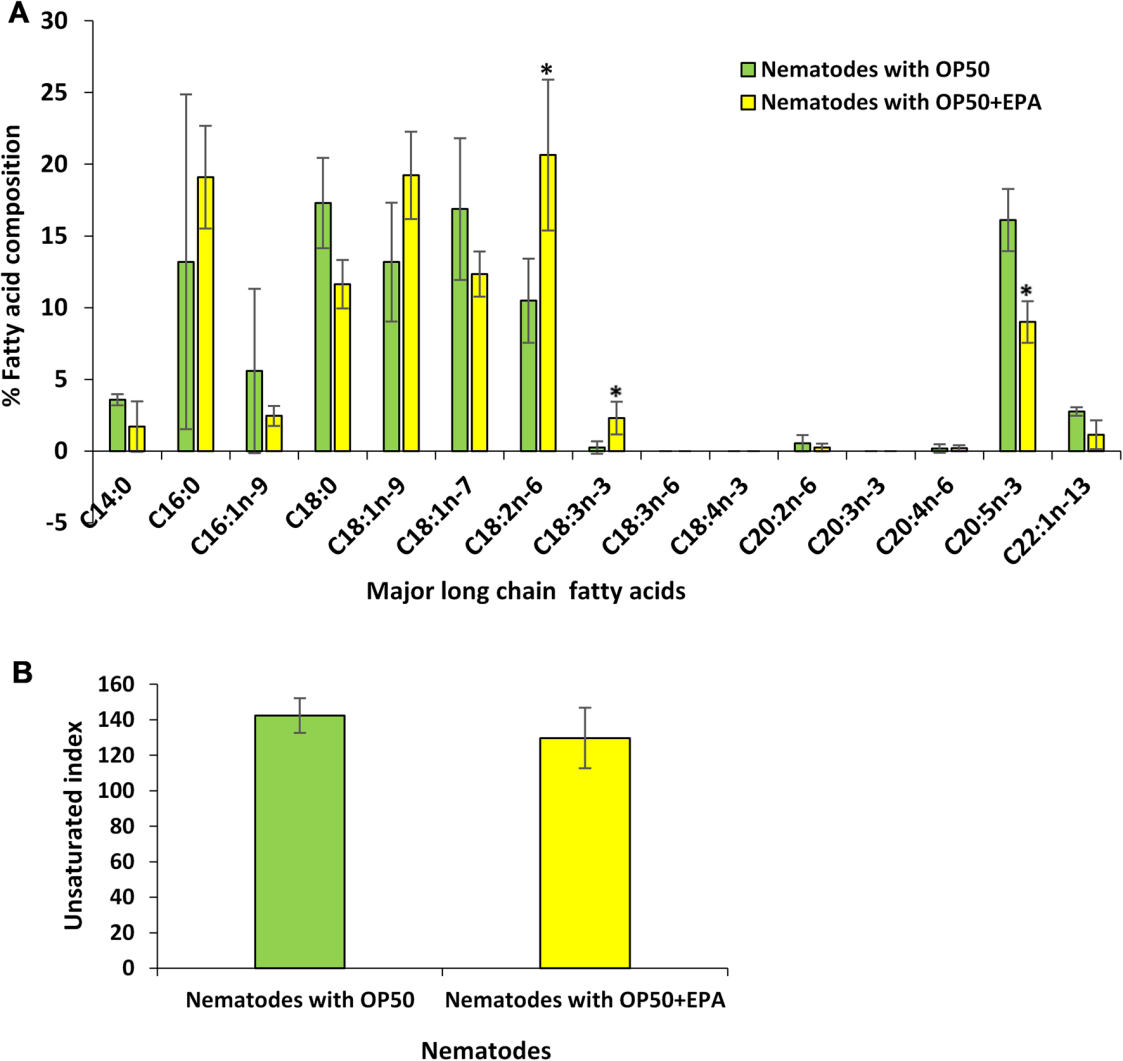
Supplementation of eicosapentaenoic acid (EPA) changes the fatty acid composition of major long-chain fatty acids in the nematodes. **A** Effect of EPA supplementation on fatty acid composition of major long-chain fatty acids of *Caenorhabditis* *elegans* with *Escherichia* *coli* OP50. Values represent the mean of three independent experiments and error bars represents the standard deviations. Asterisk (*) indicate *P* < 0.05 compared to unsupplemented nematodes. **B** Effect of EPA supplementation on unsaturation index of *C.* *elegans* on *E.* *coli* OP50

The effect of EPA supplementation on the fatty acid profile of nematodes grown on their standard laboratory food source, *Escherichia* *coli* OP50, is depicted in Fig. 1. Surprisingly, supplementation caused a significant decrease in the percentage of EPA, with a concomitant increase in the percentage of 18:2n-6 (*P* < 0.005) and α-linolenic acid (18:3n-3) (*P* < 0.005) compared to unsupplemented nematodes (Fig. 1A). In order to better understand these results, the unsaturation indexes of *C.* *elegans* lipids were calculated and it was found that these changes in fatty acid composition allowed *C.* *elegans* to maintain its normal unsaturation index despite supplementation with a PUFA (Fig. 1B). In order to verify these results, we studied the relative expression of genes involved in fatty acid metabolism and found that several genes involved in lipid metabolism, including *fat* genes (*fat-2*, *fat-5* and *fat-6*), *elo* genes (*elo-3*, *elo-7* and *elo-8*), and cytochrome P450 genes (*cyp-29A3*, *cyp-33C1* and *cyp-33E1*) were significantly up-regulated (Table 1).

**Table 1:** *Caenorhabditis* *elegans* genes differentially expressed by eicosapentaenoic acid supplementation

Gene expression	Genes		Fold change	*P* value	Lower 95% CI	Upper 95% CI
Up-regulated	Lipid metabolism	*cyp-14A2*	6.13	0.0099	1.77	21.21
		*cyp-29A3*	6.03	0.0073	2.33	15.57
		*cyp-33C1*	5.05	0.0245	1.19	21.39
		*cyp-33E1*	4.91	0.0123	1.53	15.84
		*cyp-37B1*	4.96	0.0150	1.44	17.05
		*elo-3*	5.14	0.0195	1.18	22.32
		*elo-7*	5.16	0.0076	2.08	12.78
		*elo-8*	4.8	0.0092	1.72	13.44
		*fat-2*	1.66	0.0094	1.2	2.29
		*fat-5*	3.36	0.0073	1.8	6.26
		*fat-6*	2.4	0.0161	1.24	4.62
	Immune response	*abf-2*	5.58	0.0129	1.9	16.36
		*abf-3*	4.5	0.0112	1.59	12.74
		*cht-1*	5.99	0.0051	2.17	16.49
		*cnc-4*	3.11	0.0445	1.07	9.02
		*col-179*	2.63	0.0407	0.96	7.23
		*daf-16*	5.64	0.0369	0.76	41.71
		*emb-8*	4.36	0.0172	1.34	14.24
		*fipr-22*	2.92	0.0242	1.41	6.09
		*ilys-2*	4.68	0.0192	1.34	16.38
		*lys-5*	4.24	0.0193	1.4	12.88
		*lys-6*	4.51	0.0337	1.12	18.22
		*mboa-7*	5.95	0.0025	2.72	13.01
		*nhr-49*	5.11	0.0081	1.82	14.39
		*spp-12*	6.29	0.0074	2.26	17.55
Down-regulated	None					

The table represents genes with a fold change of ≥ 1.5. *P* values ≤ 0.05 indicate a significant difference from control (unsupplemented nematodes with *E.* *coli* OP50), with lower and upper percentage confidence intervals (CI)

**Fig. 2:**
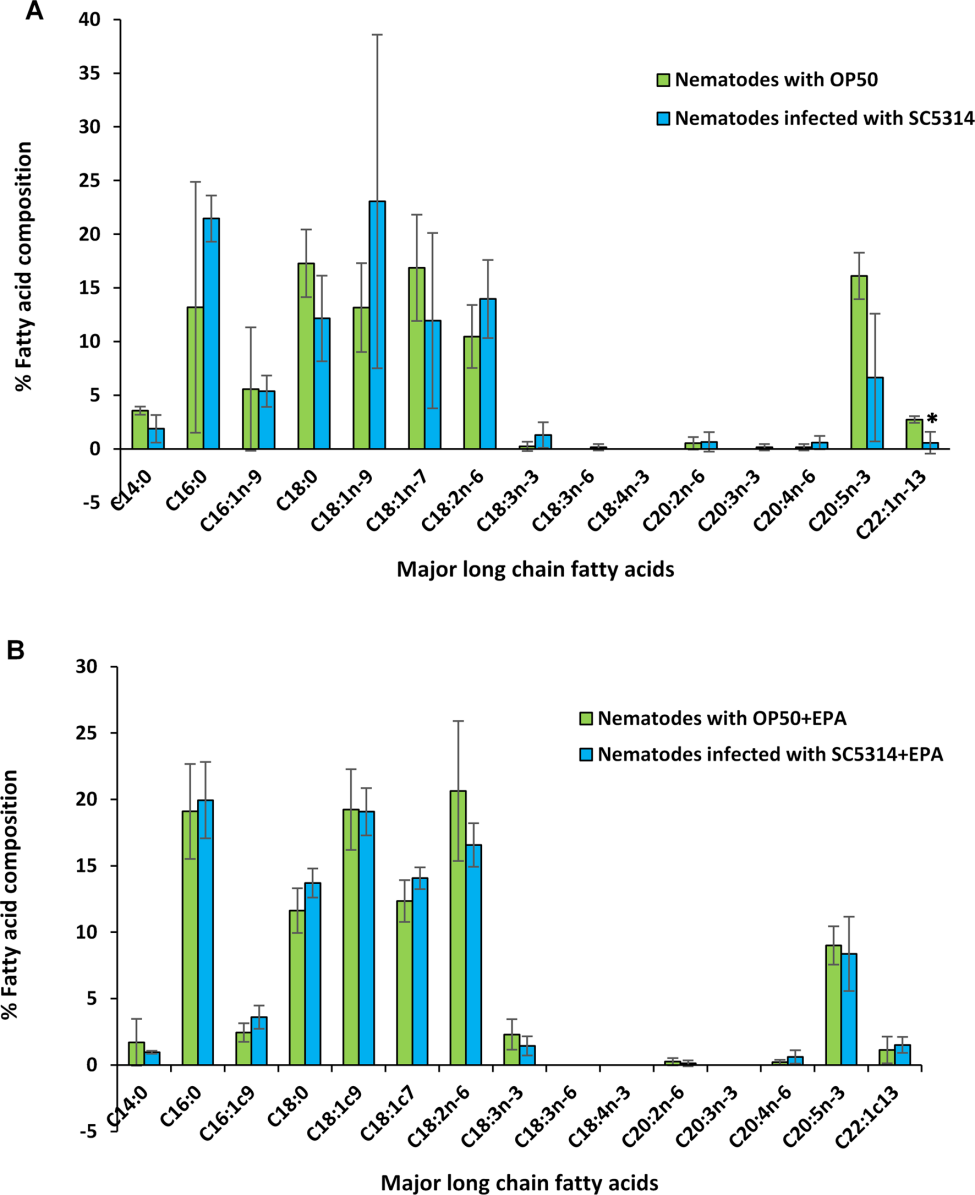
Infection by *Candida* *albicans* did not cause any significant change in fatty acid composition. Effect of *C.* *albicans* SC5314 infection on fatty acid profiles of **A** unsupplemented nematodes and **B** nematodes supplemented with eicosapentaenoic acid (EPA). Values represent the mean of three independent experiments and error bars represent the standard deviations. Asterisk (*) indicates *P* < 0.05 compared to uninfected nematodes with *Escherichia* *coli* OP50

We also examined the influence of *C.* *albicans* infection on fatty acid profiles of unsupplemented nematodes and nematodes supplemented with EPA. However, both supplemented and unsupplemented nematodes had similar fatty acid profiles to their respective uninfected controls, indicating that infection by *C.* *albicans* did not cause any significant change in fatty acid composition (Fig. 2). In addition, none of the genes involved in fatty acid synthesis was significantly regulated due to infection in unsupplemented nematodes (Table 2). However, infection of EPA-supplemented nematodes resulted in up-regulation of *fat-1* and significant down-regulation of lipid metabolism genes, including *fat* genes *fat-5*, *elo-5*, *elo-7*, *cyp-29A3*, *cyp-33C1* and *cyp-33E1* (Table 2). This may negate the effect of EPA supplementation seen in Table 1, resulting in no change in the fatty acid composition.

**Table 2:** *Caenorhabditis* *elegans* genes differentially expressed by eicosapentaenoic acid supplementation during *Candida* *albicans* infection

Conditions	Gene expression	Genes		Fold change	*P* value	Lower 95% CI	Upper 95% CI
*C.* *albicans* vs *E.* *coli* OP50	Up-regulated	Immune response	*col-179*	1.67	0.0495	0.99	2.81
	Down-regulated	Immune response	*ilys-5*	− 1.84	0.0400	0.3	− 1.02
			*lys-4*	− 3.32	0.0151	0.13	− 1.41
			*spp-2*	− 2.51	0.0116	0.21	− 1.29
			*spp-14*	− 1.69	0.0047	0.45	− 1.27
*C.* *albicans* + EPA vs *E.* *coli* OP50 + EPA	Up-regulated	Lipid metabolism	*cyp-29A2*	3.25	0.0280	1.24	8.48
			*elo-9*	2.8	0.0264	1.09	7.16
			*fat-1*	2.45	0.0434	0.97	6.22
	Down-regulated	Lipid metabolism	*cyp-29A3*	− 4.9	0.0252	0.07	− 1.62
			*cyp-33C1*	− 3.97	0.0483	0.07	− 1.03
			*cyp-33E1*	− 4.81	0.0261	0.07	− 1.58
			*cyp-37B1*	− 4.84	0.0254	0.07	− 1.61
			*elo-5*	− 14.2	0.0013	0.05	− 9.47
			*elo-7*	− 4.2	0.0273	0.08	− 1.48
			*fat-5*	− 3.29	0.0064	0.2	− 2.18
		Immune response	*cht-1*	− 1.82	0.0372	0.33	− 1.09
			*ilys-5*	− 5.29	0.0033	0.12	− 3.49
			*lys-5*	− 4.37	0.0164	0.1	− 1.92
			*lys-6*	− 6.13	0.0169	0.06	− 2.2
			*nhr-49*	− 4.65	0.0175	0.09	− 1.92
			*spp-12*	− 5.24	0.0257	0.06	− 1.63

The table represents genes with a fold change of ≥ 1.5. *P* values ≤ 0.05 indicate a significant difference from uninfected nematodes with lower and upper percentage confidence intervals (CI)

### EPA supplementation influences survival of *C. albicans*-infected nematodes by inhibiting hyphal formation

**Fig. 3:**
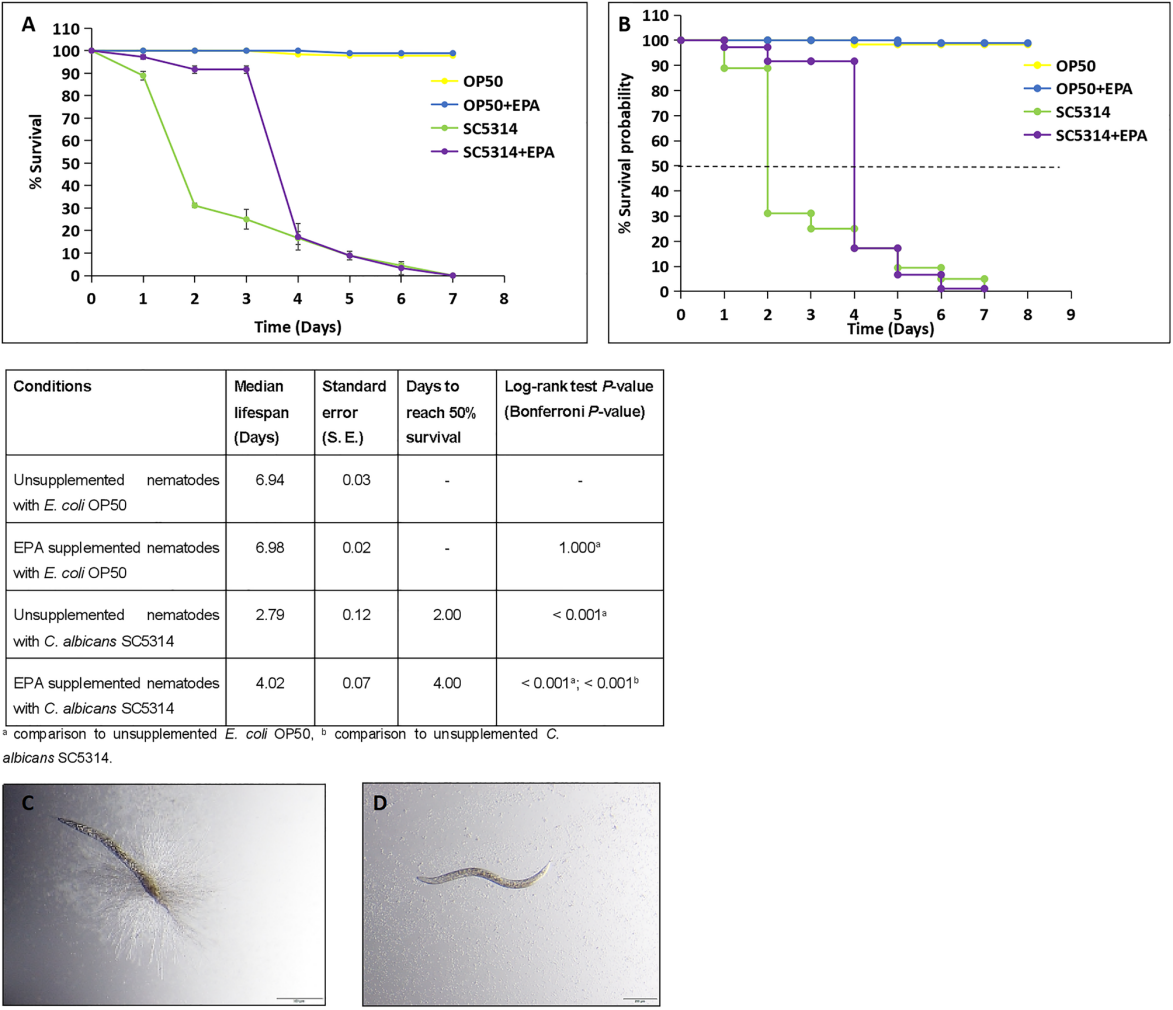
Eicosapentaenoic acid (EPA) supplementation influences survival of *Candida* *albicans*-infected nematodes by inhibiting hyphal formation. **A** *Candida* *albicans* killing of *Caenorhabditis* *elegans* supplemented with EPA, compared to unsupplemented nematodes. Data points are the average of three independent experiments and error bars indicate the standard deviations. **B** Kaplan–Meier graphs indicating the survival probability of *C.* *elegans*. The survival of nematodes with or without EPA was significantly reduced when exposed to *C.* *albicans* SC5314 compared to *Escherichia* *coli* OP50 control (*P* < 0.05). Unsupplemented nematodes are more susceptible to killing by *C.* *albicans* (*P* < 0.01). The EPA supplementation significantly rescued the initial susceptibility to pathogen within 4 days of infection. Dotted line represents 50% killing of *C.* *elegans*. The table represents median lifespan with standard error (S. E.) along with days to reach 50% mortality. Bonferroni *P* values are included for the Log-rank test for overall differences in survival. **C** A micrograph of an unsupplemented nematode, infected with *C.* *albicans* SC5314 indicating hyphal production at day 2 of infection. **D** A micrograph of a EPA-supplemented nematode, infected with *C.* *albicans* SC5314 at day 2 of infection. None of the EPA-supplemented nematodes showed any hyphal formation. Scale bar represent 200 µm

To elucidate the role of PUFAs in *C.* *elegans* response to *C.* *albicans*, nematodes were raised in the presence of dietary EPA and infected with the yeast. This experiment was performed in triplicate and reproducible differences were observed between supplemented and unsupplemented infected nematodes (Fig. 3A). Figure 3B shows the results and statistical analysis of the completed lifespan assays. Unsupplemented nematodes were more susceptible to killing by *C.* *albicans* (*P* < 0.01), with infection causing death in 50% of the nematodes after 2 days and 100% mortality after 7 days. In addition, as also reported by Pukkila–Worley and co-workers [40], during the first 2 days of infection, hyphal production was observed in some unsupplemented *C.* *albicans-*infected nematodes (Fig. 3B). However, after this initial 2-day period, a slower killing phase was observed in unsupplemented *C.* *albicans*-infected nematodes with the absence of hyphae in any of these infected nematodes.

EPA supplementation, prior to infection with *C.* *albicans*, significantly rescued the initial susceptibility to pathogen during the first 3 days (*P* < 0.01) (Fig. 3A, B), although it did not influence the time needed to kill 100% of the nematodes. Strikingly, no hyphal formation was observed in any of the EPA-supplemented nematodes infected with *C.* *albicans* (Fig. 3C) at any time point during the experiment, suggesting that EPA supplementation inhibited the yeast to hyphal conversion in the nematodes, preventing the initial hyphal-mediated killing of the nematodes.

### Exogenous 17,18-EpETE inhibits hyphal formation in vitro and in vivo as well as survival

**Fig. 4:**
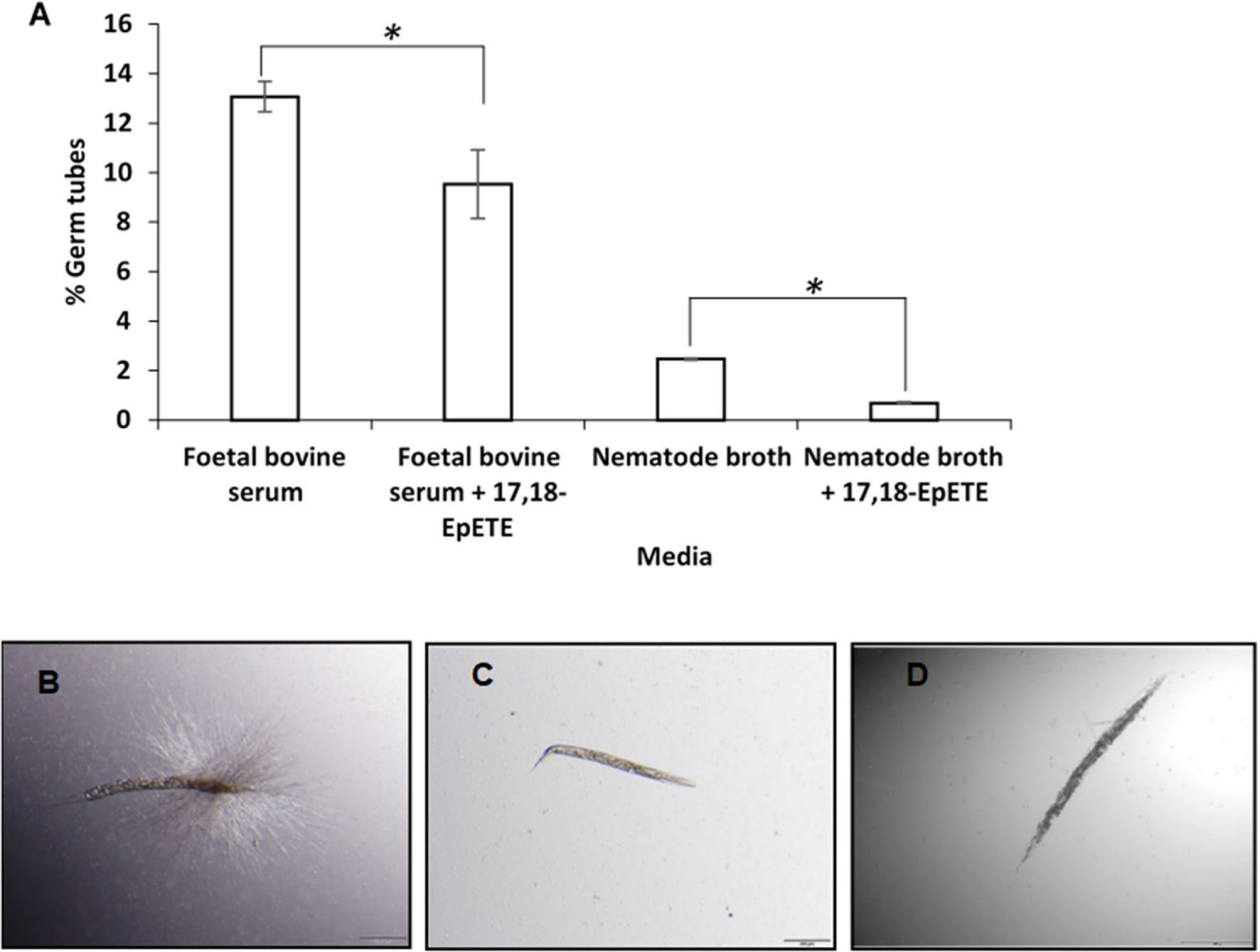
Exogenous 17,18-epoxyeicosatetraenoic acid (17,18-EpETE) inhibits hyphal formation in vitro and in vivo. **A** Effect of 17,18-EpETE on germ tube formation of *Candida* *albicans.* Values represents the mean of three independent experiments and error bars represent the standard deviations. Asterisk (*) indicate *P* < 0.05 compared to 17,18-EpETE unsupplemented media. **B** *C.* *albicans* SC5314-infected control nematodes with hyphal production. **C** Eicosapentaenoic acid (EPA) supplemented *C.* *albicans* SC5314-infected nematodes with no hyphal production. **D** 17,18-EpETE supplemented *C.* *albicans* SC5314-infected nematodes with no hyphal production. Scale bars represent 200 µm

**Fig. 5:**
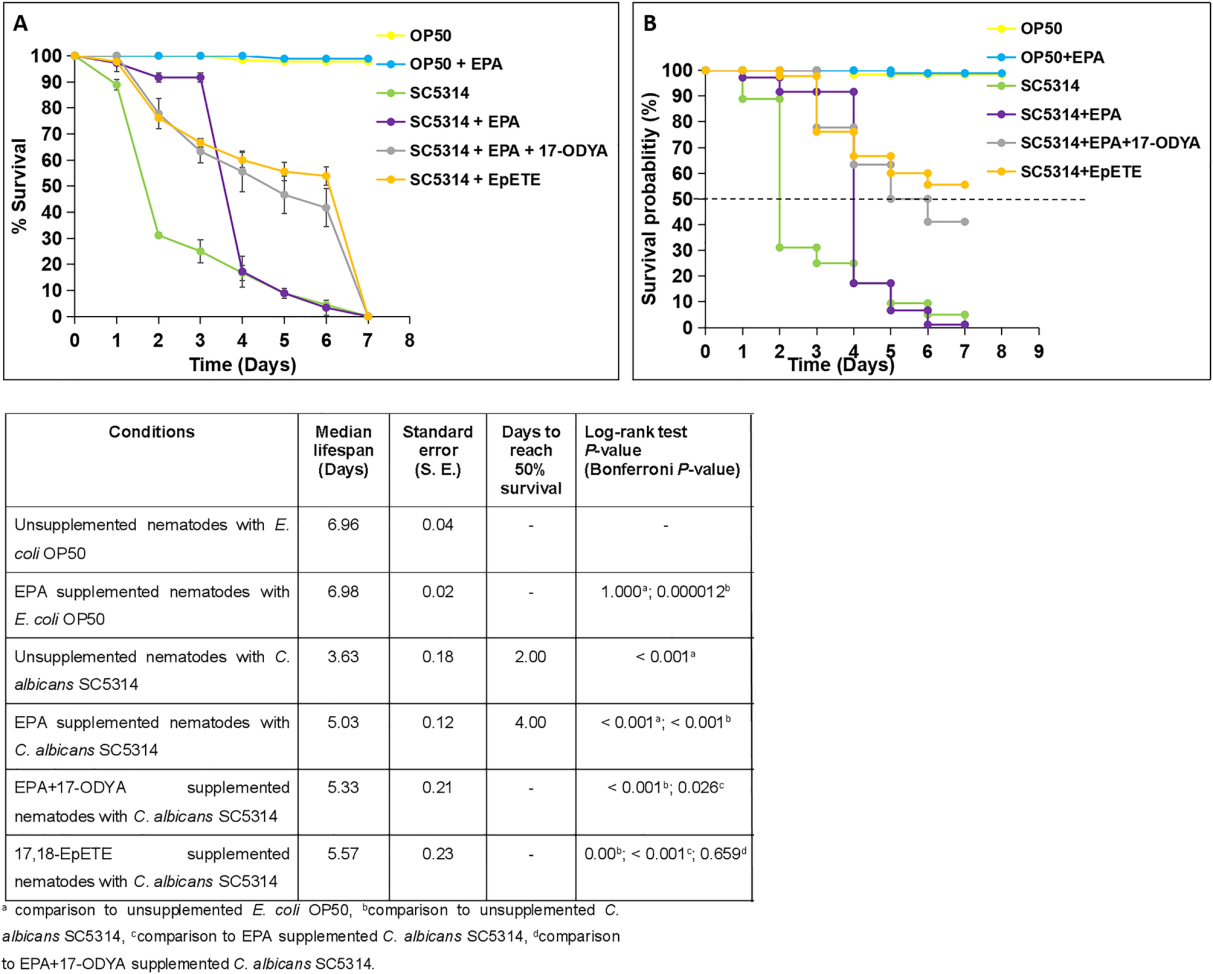
17,18-Epoxyeicosatetraenoic acid (17,18-EpETE) supplementation or exposure to 17-octadecynoic acid (17-ODYA) influences survival of *Candida* *albicans*-infected nematodes. **A** *Candida* *albicans* killing of *Caenorhabditis* *elegans* supplemented with 17,18-EpETE or 17-ODYA, compared to unsupplemented nematodes and nematodes supplemented with eicosapentaenoic acid (EPA). Data points are the average of three independent experiments and error bars indicate the standard deviations. **B** Kaplan–Meyer graphs indicating the survival probability of *C.* *elegans*. The survival of nematodes with 17,18-EpETE was significantly increased, while inhibition of the conversion of EPA to 17,18-EpETE by the addition of 17-ODYA partially restored virulence. Dotted line represents 50% killing of *C.* *elegans*. The table represents median lifespan with standard error (S. E.) along with days to reach 50% mortality. Bonferroni *P* values are included for the Log-rank test for overall differences in survival

Given the observed decrease in the percentage EPA in the supplemented nematodes and the fact that EPA is known not to be able to influence hyphal formation [41], we hypothesised that it is unlikely that EPA supplementation directly affects in vivo hyphal production by *C.* *albicans,* but rather that its influence is indirect, possibly via in vivo produced eicosanoid products of EPA, such as 17,18-EpETE, which is the most predominant cytochrome (CYP) eicosanoid in *C.* *elegans* [37, 38]. Therefore, to further analyse the link between long-chain PUFAs, CYP eicosanoids, and the observed *C.* *albicans* morphological changes, we investigated 17,18-EpETE for its ability to inhibit hyphal formation both in vitro and in vivo in *C.* *elegans*. First, we tested this hypothesis by exposing *C.* *albicans* to 17,18-EpETE in hyphal inducing media. As depicted in Fig. 4A, the addition of 17,18-EpETE to *C.* *albicans* yeast cells in either foetal bovine serum or nematode broth caused a significant decrease in germ tube formation, compared to control cells. Similar results were also observed for a crystal violet germ tube assay (Figure S1), indicating the possibility that production of this eicosanoid from EPA may explain the reduced hyphal formation in *C.* *elegans*. To gain further insight into the role of 17,18-EpETE in *C.* *albicans* hyphal formation in vivo, nematodes infected with *C.* *albicans* yeast cells, were supplemented with 17,18-EpETE, while unsupplemented nematodes were used as control (Fig. 4B). Similar to EPA-supplemented nematodes, infected with *C.* *albicans* (Fig. 4C), no hyphal formation was observed in 17,18-EpETE supplemented nematodes infected with *C.* *albicans* during the first 24 h post infection (Fig. 4D). Thus, revealing that 17,18-EpETE was able to inhibit hyphal formation of *C.* *albicans* in vivo as well (Fig. 4A). In order to determine if hyphal inhibition by 17,18-EpETE may influence the ability of *C.* *albicans* to kill the nematodes (as was seen during initial infection by EPA-supplemented nematodes), the survival of these 17,18-EpETE-supplemented nematodes was determined. As can be seen from Fig. 5, supplementation with 17,18-EpETE significantly increased the survival of the infected nematodes and it was observed that none of the 17,18-EpETE-supplemented infected nematodes exhibited any hyphae able to pierce the cuticle, similar to EPA-supplemented nematodes. Although the reason for the difference observed between the survival of the EPA- and 17,18-EpETE-supplemented nematodes is not known, it may be due to a concentration effect as the amount of EPA converted to 17,18-EpETE (during EPA supplementation) may be less than the concentration available during 17,18-EpETE supplementation.

### Exogenous cytochrome P450 inhibitors restore hyphal formation of *C. albicans* in vivo

**Fig. 6:**
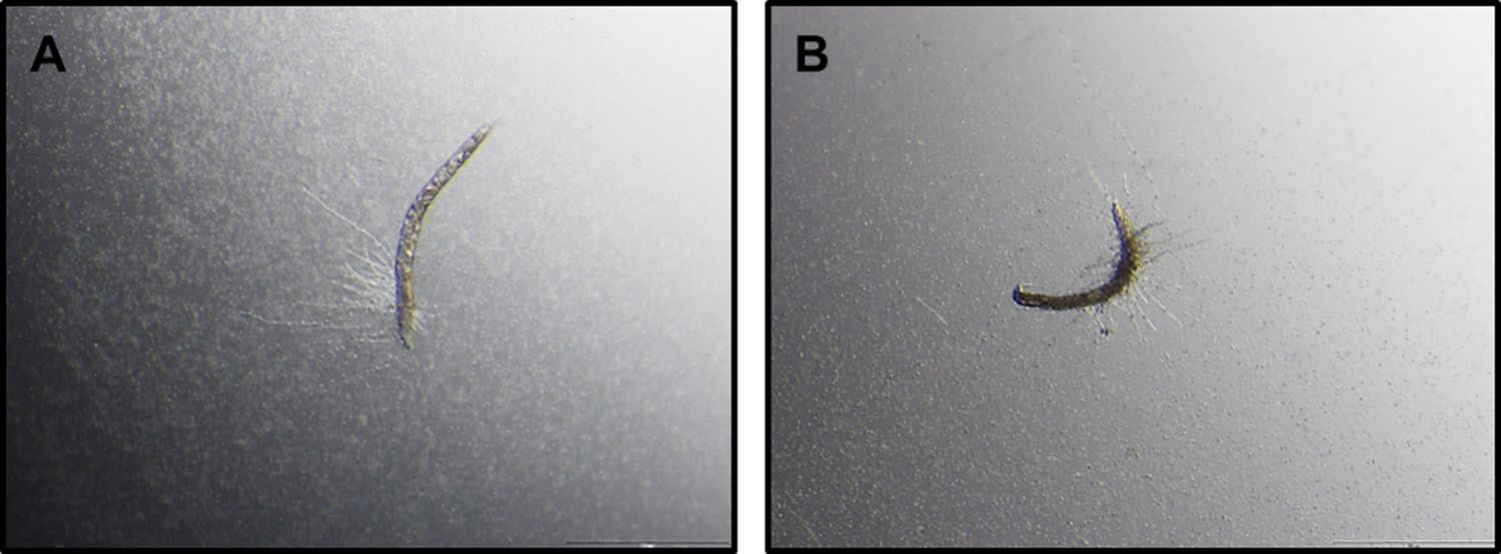
Exogenous cytochrome P450 inhibitors restore hyphal formation of *Candida* *albicans* in vivo. **A** A micrograph of a representative of eicosapentaenoic acid (EPA)-supplemented *C.* *albicans*-infected nematodes treated with 6-(2-propargyloxyphenyl)hexanoic acid (PPOH) taken at day 2 of infection. **B** A micrograph of a representative of EPA-supplemented *C*. *albicans*-infected nematodes treated with 17-octadecynoic acid (17-ODYA) taken at day 2 of infection. Scale bars represent 200 µm

In order to confirm the role of CYP derived 17,18-EpETE, we tested the hypothesis that CYP inhibitors, 17-octadecynoic acid (17-ODYA) and 6-(2-propargyloxyphenyl)hexanoic acid (PPOH) (two compounds mostly used to block mammalian CYP isoforms involved in EPA metabolism), will inhibit the *C.* *elegans* CYP450 activity, responsible for epoxidation of EPA to 17,18-EpETE, and restore *C.* *albicans* hyphal formation. In agreement with this hypothesis, the nematodes that were pre-treated with either PPOH (Fig. 6A) or 17-ODYA (Fig. 6B) thereafter infected with *C.* *albicans*, showed a partial restoration of *C.* *albicans* hyphal formation.

Interestingly, exposure of EPA-supplemented nematodes to the CYP inhibitor, 17-ODYA, did not restore the survival to unsupplemented nematode levels (Fig. 5). Although it is known that 17-ODYA does not to influence growth or morphology of *C.* *albicans*, it can interfere with the yeast’s ability to produce bioactive oxidised lipids, presumably by inhibiting *C.* *albicans* CYPs [42]. Therefore, additional as yet unknown effects of this inhibitor on both *C.* *albicans* and *C.* *elegans* may influence the outcome of infections in this model.

### EPA supplementation affects *C. albicans* gene expression in vivo

Since EPA supplementation inhibited hyphal formation of *C*. *albicans* in vivo, we investigated the gene expression changes elicited by EPA on *C*. *albicans*, with a focus on genes involved in morphogenesis. The differentially expressed genes in response to EPA are given in Table 3, containing 112 genes (74 up-regulated and 38 down-regulated). Among the up-regulated genes, 64 genes are involved in hyphal formation (Supplementary Table S1).

**Table 3:** *Candida* *albicans* genes differentially expressed by eicosapentaenoic acid in vivo

Conditions	Genes		Fold change	*P* value	Lower 95% CI	Upper 95% CI
Up-regulated	Filamentation	*ACE2*	3.83	0.0003	2.93	5
		*ADR1*	2.96	0.0087	1.47	5.99
		*AFT2*	3.26	0.0056	1.63	6.53
		*CAS5*	2.66	0.0047	1.6	4.41
		*CPH1*	3.6	0.00002	3.5	3.7
		*CRZ1*	3.23	0.0025	1.85	5.64
		*CSR1*	4.25	0.0013	3.39	5.32
		*CTA4*	2.16	0.0023	1.6	2.92
		*CTA8*	1.84	0.0186	1.2	2.82
		*CWT1*	2.42	0.0104	1.48	3.95
		*ERG11*	2.14	0.0288	1.2	3.8
		*FCR1*	1.62	0.0061	1.23	2.14
		*FGR13*	2.77	0.0050	2.01	3.81
		*FGR17*	3.18	0.0001	2.64	3.83
		*FGR27*	2.49	0.0229	1.13	5.53
		*FKH2*	2.93	0.0039	1.73	4.95
		*HAP5*	3.95	0.0001	3.06	5.1
		*HWP1*	3.63	0.00009	2.89	4.56
		*LSC2*	2.02	0.0005	1.7	2.39
		*MDR1*	2.7	0.0295	1.27	5.71
		*NGS1*	3.4	0.0335	1.17	9.9
		*NOT3*	3.35	0.0006	2.95	3.82
		*OPI1*	2.19	0.0205	1.15	4.17
		*PPR1*	3.12	0.0017	2.02	4.82
		*RBF1*	3.15	0.0054	1.74	5.7
		*RCA1*	1.93	0.0064	1.35	2.77
		*RFX2*	3.12	0.0044	2.24	4.33
		*RLM1*	3.4	0.0010	2.17	5.32
		*RON1*	4.63	0.0096	2.41	8.9
		*RTG3*	2.39	0.0128	1.26	4.54
		*SAP6*	4.15	0.0184	1.78	9.65
		*SET3*	2.66	0.0027	1.83	3.86
		*SFL2*	1.93	0.0265	1.08	3.47
		*SIN3*	2.88	0.0044	1.73	4.8
		*SKN7*	3.16	0.0159	1.25	7.96
		*SKO1*	2.25	0.0469	1.02	4.97
		*SNF4*	1.58	0.0385	1.04	2.41
		*SNF5*	2.92	0.0197	1.22	6.96
		*SNF6*	3.29	0.0065	1.62	6.67
		*SNQ2*	3.12	0.00009	2.53	3.85
		*SPT3*	2.79	0.0045	1.8	4.33
		*SPT6*	2.79	0.0045	1.8	4.33
		*SPT20*	3.16	0.0051	1.7	5.87
		*STD1*	2.15	0.0182	1.32	3.49
		*SWI1*	3.14	0.0092	1.67	5.91
		*SWI4*	2.83	0.0154	1.28	6.26
		*TAC1*	2.6	0.0350	1.01	6.74
		*TEC1*	2.12	0.0184	1.14	3.97
		*TFG1*	1.86	0.0191	1.11	3.14
		*TUP1*	1.89	0.0114	1.36	2.61
		*UME6*	3.67	0.0137	1.89	7.12
		*WOR1*	2.7	0.0403	1.11	6.53
		*YOR1*	1.8	0.0359	1.07	3.04
		*ZCF3*	3.09	0.0076	1.69	5.65
		*ZCF7*	3.18	0.0060	2.15	4.7
		*ZCF11*	3.4	0.0018	2.16	5.34
		*ZCF14*	3.05	0.0327	1.09	8.54
		*ZCF17*	3.08	0.0006	2.15	4.4
		*ZCF18*	3.02	0.0071	1.74	5.25
		*ZCF29*	2.86	0.0020	1.79	4.59
		*ZCF32*	3.7	0.0005	2.86	4.79
		*DAL8*	4.62	0.0002	3.17	6.73
	Other processes (Table S1)	*EHT1*	2.36	0.0189	1.21	4.61
		*ECM17*	2.59	0.0255	0.97	6.97
		*FAH2*	2.83	0.0014	1.85	4.32
		*FLU1*	2.57	0.0018	2.16	3.06
		*HRD3*	1.99	0.0030	1.53	2.59
		*IPT1*	2.79	0.0064	1.95	3.99
		*MET4*	2.84	0.0116	1.55	5.18
		*PST1*	3.94	0.0005	2.61	5.95
		*RGT1*	2.36	0.0203	1.07	5.19
		*RTA3*	3.77	0.0007	2.58	5.53
		*SUL2*	2.98	0.0156	1.65	5.41
		*SUT1*	2.53	0.0132	1.27	5.03
Down-regulated	Filamentation	*ALS1*	− 2.94	0.0010	0.23	− 1.98
		*CPH2*	− 1.92	0.0026	0.4	− 1.48
		*CUP9*	− 3.01	0.0005	0.24	− 2.15
		*RIM101*	− 2.64	0.0015	0.26	− 1.8
		*STP2*	− 1.69	0.0253	0.38	− 1.08
		*TDH3*	− 1.95	0.00004	0.46	− 1.76
		*TYE7*	− 2.87	0.0017	0.22	− 1.85
	Other processes (Table S1)	*YWP1*	− 1.97	0.0078	0.37	− 1.42

The table represents genes with a fold change of ≥ 1.5. *P* values ≤ 0.05 indicate a significant difference from unsupplemented *C.* *albicans*-infected nematodes, with lower and upper percentage confidence intervals (CI)

### EPA supplementation primes *C*. *elegans* immune response

We determined changes in the expression of genes involved in defence against infection by comparing the EPA-supplemented nematodes with the unsupplemented control nematodes. Among the up-regulated genes, we observed several genes with potential roles in detoxification or antimicrobial activities (*abf-2*, *abf-3*, *cht-1*, *cyp-14A2*, *cnc-4* and *col-179*) (Table 1), consistent with their involvement in a protective host response (Supplementary Table S1). Other immune response genes that were up-regulated include *cyp-37B1*, *daf-16*, *fipr-22*, *ilys-2*, *lys-5*, *lys-6*, *mboa-7* and *spp-12* (Table 1). Moreover, among these up-regulated genes observed in EPA-supplemented nematodes was *nhr-49*, which is involved in the regulation of fatty acid metabolic processes, determination of adult lifespan, immuno-metabolic response to bacterial infection and positive transcriptional regulation from RNA polymerase II promoter in response to stress [43]. In addition to its function in lipid metabolism regulation, *nhr-49* also plays a vital role in a cytoprotective acute stress response programme that functions independently and parallel of HLH-30/TFEB and SKN-1/Nrf2 signalling [44–46].

In order to determine the effect of EPA supplementation on the immune response to *C.* *albicans* infection, we compared the gene expression of EPA-supplemented nematodes infected with *C.* *albicans* to unsupplemented *C.* *albicans*-infected nematodes. Several genes encoding CYPs were significantly (*P* ≤ 0.05) up-regulated, including *cyp-29A2* and *cyp-37A1* (Table 4), which are expressed in the intestine and functions in lipid storage and life span [47] as well as *cyp-14A2*, which is involved in stress response and detoxification [48]. Furthermore, it is known that the host response towards *C.* *albicans* includes induction of specific defences and common immune genes [49]. Since EPA supplementation prior to infection with *C.* *albicans*, significantly extended the time needed to kill 50% of the nematodes compared to unsupplemented infected nematodes (Fig. 3), the effect of EPA supplementation on the host immune response to infection was also investigated. Interestingly, among the effector genes significantly up-regulated (*P* ≤ 0.05) in EPA-supplemented nematodes infected with *C.* *albicans*, compared to unsupplemented infected nematode*s* (Table 4), were those that had previously been demonstrated to be involved in *C.* *elegans* immune response to bacteria, such as C-type lectin genes, *clec-60* and *clec-67* [50–52] as well as caenopore genes, *spp-2* (involved in defence response to Gram-positive bacteria) and *spp-14* (involved in immune response) (Table 4). Under this condition, *fat-3* was also up-regulated. Nandakumar and Tan [53] found that *fat-3* regulates the expression of stress response and infection genes, involved in immune function and oxidative stress response.

**Table 4:** *Caenorhabditis* *elegans* genes differentially expressed by eicosapentaenoic acid supplementation during *Candida* *albicans* infection

Gene expression	Genes		Fold change	*P* value	Lower 95% CI	Upper 95% CI
Up-regulated	Lipid metabolism	*cyp-14A2*	4.15	0.0305	1.39	12.4
		*cyp-29A2*	5.15	0.0020	2.52	10.53
		*cyp-37A1*	5.05	0.0244	1.67	15.33
		*elo-6*	1.98	0.0306	1.03	3.81
		*elo-9*	6.49	0.0022	2.75	15.3
		*fat-3*	1.87	0.0413	0.92	3.8
		*fat-4*	2.4	0.0039	1.61	3.59
		*fat-6*	2.22	0.0339	1.11	4.46
	Immune response	*clec-60*	2.35	0.0425	1.02	5.41
		*clec-67*	4.84	0.0247	1.63	14.37
		*spp-2*	3.44	0.0038	1.85	6.4
		*spp-14*	1.76	0.0018	1.4	2.22
Down-regulated	Lipid metabolism	*elo-5*	− 1.82	0.0372	0.33	− 1.09

The table represents genes with a fold change of ≥ 1.5. *P* values ≤ 0.05 indicate a significant difference from unsupplemented *C.* *albicans*-infected nematodes with lower and upper percentage confidence intervals (CI)

## Discussion

**Fig. 7:**
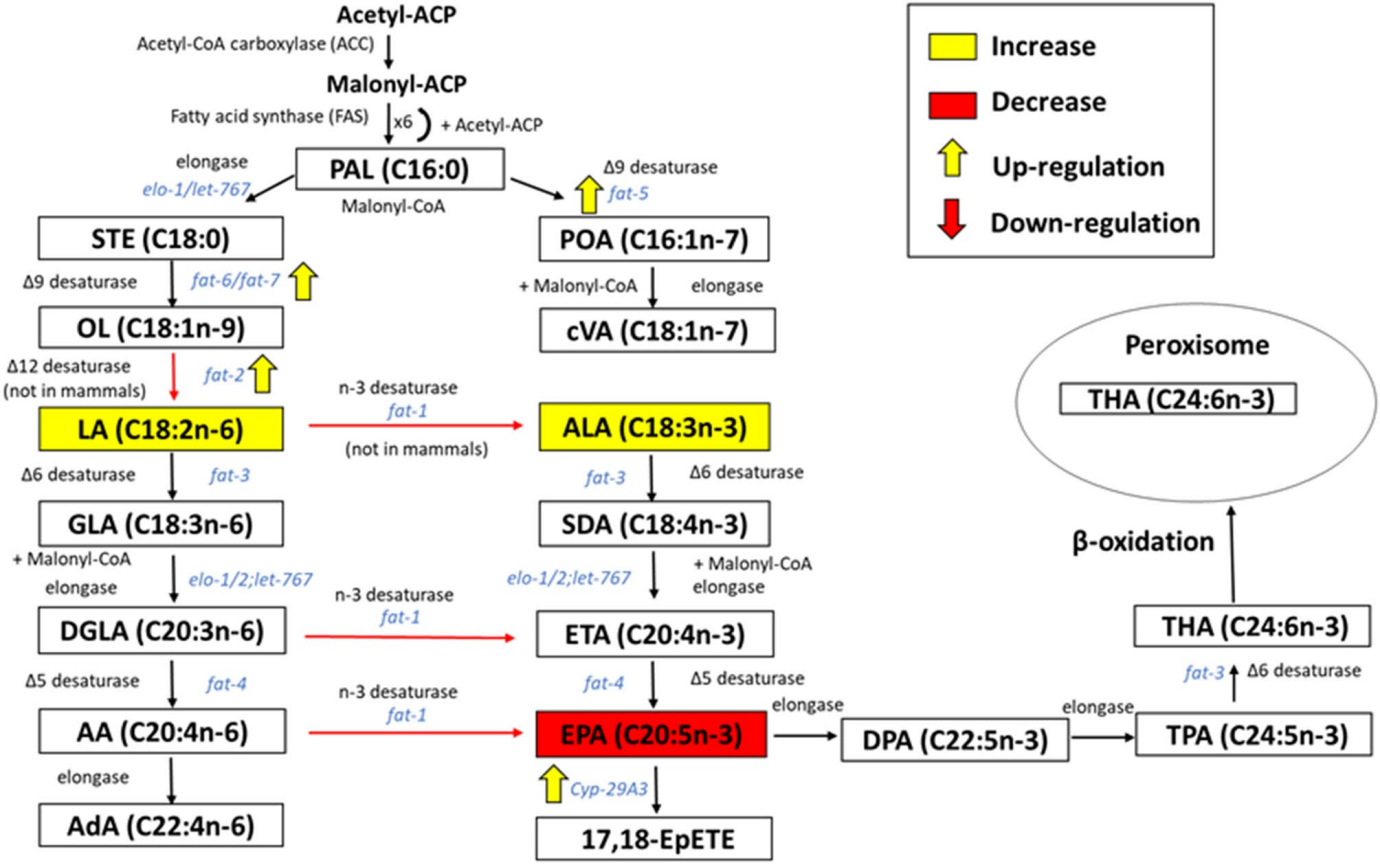
Effect of eicosapentaenoic acid on *Caenorhabditis* *elegans* polyunsaturated fatty acid biosynthesis pathway. Yellow squares indicate significant increase, while red squares indicate significant decrease in percentage of fatty acids compared to unsupplemented nematodes. Yellow arrows indicate up-regulation, while red arrows indicate down-regulation of genes compared to unsupplemented nematodes

*Caenorhabditis elegans* is one of the adaptable model organisms which offers many experimentally desirable traits and can be used to address questions that may lead to finding potential antimicrobial drugs or targets. Fatty acid supplementation is one of the effective ways to manipulate or alter the fatty acid composition of nematodes; moreover, it can also be used in rescuing defects in fatty acid-deficient mutants [27]. Fatty acid metabolism is known to play a significant role in many physiological and pathological processes [54] and research has shown that manipulating lipid metabolism through various dietary fatty acid supplementations could extend *C*. *elegans* lifespan [55–57]. Previous studies have also demonstrated that fatty acid supplementation may have diverse roles in susceptibility to pathogenic bacteria [53, 54, 58, 59]. Thus, the alteration of *C*. *elegans* fatty acid composition may influence the host to mount a series of protective defence responses. Given this background, we examined the influence of dietary supplementation with EPA, the most predominant PUFA in *C*. *elegans* [37], on the susceptibility of *C*. *elegans* to infection by the pathogenic yeast, *C.* *albicans*. Surprisingly, EPA supplementation did not cause an increase in the fatty acid percentage of EPA in the total lipids of *C*. *elegans* (Fig. 1), but modulated the expression of lipid metabolising genes, including up-regulating those encoding for a CYP responsible for metabolising EPA to eicosanoids (Table 1). This was consistent with results observed by Bouyanfif [60], where supplementation with EPA did not cause any significant change in EPA in wild-type nematodes, although a decreasing trend was seen for EPA as well as a non-significant increase in 18:3n-3, similar to our results. Since EPA is the major fatty acid in *C.* *elegans*, and unregulated incorporation of such a highly unsaturated fatty acid may have detrimental effects on membrane fluidity [61], it is likely that changes in lipid metabolism would occur in order to maintain the unsaturation index within certain limits as was observed in our study. Thus, using EPA-supplemented uninfected nematodes, we studied the relative expression of genes involved in fatty acid metabolism and discovered that several genes, including *fat-2*, *fat-5*, *fat-6* and *cyp-29A3* were significantly up-regulated (Table 1). *fat-2* encodes the Δ12 desaturase enzyme, which facilitates the biosynthesis of 18:2n-6 from its substrate, 18:1n-9 [62]. Therefore, this observed up-regulation of *fat-2* corresponds to the observed increase in the percentage 18:2n-6 in EPA-supplemented nematodes. In addition, it may explain the observed increase in 18:3n-3, as the fatty acid metabolism is rerouted along this branch of the pathway (Fig. 7). The *fat-5* desaturase acts on palmitic acid (16:0) producing palmitoleic acid (16:1n-7), which can further be elongated to *cis*-vaccenic acid (18:1n-7)) [63]. It is also known that *fat-6* and *fat-7* encode desaturases which act on stearic acid (18:0) synthesising 18:1n-9 [63], and up-regulation of *fat-6* correlates with the observed decrease in the percentage 18:0 and increase in 18:1n-9, although these changes were not statistically significant. The up-regulation of *fat-5* and *fat-6* in the presence of PUFAs was also seen in a recent study by Wang and co-workers [64]. Furthermore, supplementation with EPA resulted in the up-regulation of *cyp-29A3* (Table 1), which encodes one of the two major CYPs that oxidises EPA to eicosanoids [37], such as 17,18-EpETE. This may explain the decrease percentage EPA in these nematodes.

EPA supplementation did influence the progression of infection by inhibiting the initial hyphal dependent killing in the first two days of infection as seen by us (Fig. 3) and Pukkila-Worley and co-workers [49] who reported that, during the first 48 h of infection, more than half of the nematodes died and all of these had hyphae piercing through the cuticle of the nematodes. This was followed by a second phase of slower killing by *C.* *albicans* without the production of hyphae. We, therefore, speculate that supplementation with EPA may influence the ability of *C.* *albicans* to form hyphae capable of piercing the nematode's cuticle and initiate this first rapid killing phase of the infection. It may be possible that shorter filamentous structures such as germ tubes or pseudohyphae that are not able to pierce the cuticle, may still be formed, however, we did not investigate this possibility.

In *C.* *albicans*, the yeast to hyphal transition is considered to play a vital role in the formation of biofilms and the pathogenesis of most fungal infections [65]. Inhibition of *C.* *albicans* hyphal formation by medium-, long-chain fatty acids and eicosanoids in vitro under various conditions, was previously shown [13, 16, 35, 66–68]. Similarly, Kuloyo and co-workers [69] showed that 20:4n-6 inhibits *C.* *albicans* hyphal formation. However, since in our current study, there was a decrease in the percentage EPA in the supplemented nematodes, and other in vitro studies showed that EPA had no effect on hyphal formation [41], we hypothesised that it is unlikely that EPA directly affects in vivo hyphal production by *C.* *albicans*, but rather that its influence is indirect, possibly via in vivo produced eicosanoid products of EPA, such as 17,18-EpETE. This metabolite is the most predominant CYP eicosanoid in *C.* *elegans* and is produced by CYP-13A12 [70], CYP-33E2, closely related to human CYP2J2 [38] as well as CYP-29A3 [37], and has anti-allergic and anti-inflammatory effects in some diseases of the mammalian skin and gut [71–73]. In this study, we determined the effect of 17,18-EpETE on hyphal formation in vitro by exposing *C.* *albicans* yeast cells to 17,18-EpETE in hyphal inducing media. This resulted in an inhibition of germ tube formation (Fig. 4, Figure S1). Similarly, nematodes exposed to 17,18-EpETE, prior to infection, also did not display any hyphae piercing the cuticle. Thus, revealing that 17,18-EpETE was able to inhibit hyphal formation of *C.* *albicans* in vivo. This is similar to our in vitro results; however, the complete inhibition observed in vivo suggests that there might be additional or enhanced inhibitory compounds or processes in the nematode. In order to confirm the involvement of CYP EPA metabolites, we added CYP inhibitors, 17-ODYA and PPOH, to EPA-supplemented infected nematodes. This resulted in rescue of hyphal formation (Fig. 6). Interestingly, previous studies have shown that 20:4n-6-derived eicosanoids can stimulate the yeast to hyphal transition in *C.* *albicans* in vitro [35]. This is the first report of an EPA-derived eicosanoid able to inhibit *C.* *albicans* filamentation in vitro and in vivo. The addition of exogenous 17,18-EpETE also significantly enhanced the survival of infected nematodes. Thus, this suggests that the major EPA-derived metabolite (17,18-EpETE) may contribute to the functional effects of EPA, acting as an anti-virulence agent against *C.* *albicans*.

In order to further elucidate the influence of EPA supplementation on *C.* *albicans* hyphal formation, we examined the influence of this PUFA on expression of *C.* *albicans* genes related to hyphal production and discovered that majority of these genes were up-regulated in supplemented nematodes (Table 3).

A unique profile regarding the differential regulation of transcription factors involved in the regulation of hyphal growth (either positively or negatively, depending on the conditions), were among these up-regulated genes. These are *ACE2*, *ADR1*, *AFT2*, *CAS5*, *CPH1*, *CRZ1*, *CSR1*, *CTA4*, *CTA8*, *CWT1*, *FGR17*, *FGR27*, *FKH2*, *HAP5*, *NGS1,* *NOT3,* *OPI1*, *PPR1*, *RBF1*, *RCA1*, *RFX2*, *RLM1*, *RON1*, *RLM1*, *RTG3*, *SFL2*, *SKO1*, *SPT20*, *STD1*, *TEC1*, *TUP1*, *UME6*, *ZCF3*, *ZCF7*, *ZCF11*, *ZCF14*, *ZCF17*, *ZCF18*, *ZCF29* and *ZCF32.* However, *CPH2*, *CUP9*, *RIM101* and *STP2* were down-regulated. The relatively low fold change observed may be due to the fact that only a sub-population of the yeasts in *C.* *elegans* are forming hyphae, resulting in lower population transcripts than expected if all the yeasts in unsupplemented *C.* *elegans* were forming hyphae at the specific time point studied.

Although the up-regulation of many hyphae-associated genes in the EPA-supplemented nematodes may seem counterintuitive, it must be noted that the some are negative regulators of filamentation and for many others induction of inhibition of hyphal formation may depend on the specific conditions. Up-regulated genes that are known to have both positive and negative effects on hyphal/filament formation, depending on the specific conditions, include *ADR1* [74], *AFT2* [75], *CAS5* [40], *CPH1* [76, 77], *CSR1* [74, 78], *FGR17* [74, 79], *HAP5* [80, 81], *SFL2* [82, 83], *TEC1* [83, 84], *UME6* [77, 83] and *ZCF3* [79, 83].

Interestingly, several major transcription factors were not differentially regulated. These were *ADA2*, *AHR1*, *ARG81*, *ASG1*, *ASH1*, *BCR1*, *BRE1*, *BRG1*, *CZF1*, *EFG1*, *EFH1*, *FLO8*, *GPR1*, *GRF10*, *HMS1*, *HOT1*, *MED7*, *MSS11*, *NDT80*, *NOT5*, *NRG1*, *OFI1*, *PHO4* and *RFG1*.

Similar results were seen by Kuloyo and co-workers [69], where the addition of another PUFA, 20:4n-6, caused an up-regulation of genes involved in biofilm and hyphal formation, even though exposure to this PUFA inhibited morphogenesis. This was seen as a means to compensate for the hyphal inhibitory effect of 20:4n-6. Genes involved in hyphal formation that responded similarly to EPA in vivo and 20:4n-6 in vitro are *CAS5*, *CRZ1*, *CTA4*, *ERG11*, *FCR1*, *SNQ2*, *TAC1*, *TEC1*, *YOR1* and *ZCF3*. In addition, other non-hyphal related genes up-regulated in the presence of 20:4n-6 in vitro were also up-regulated by the presence of EPA in vivo. These were *ETH1*, *FLU1*, *IPT1* and *SUT1*. This correlation between the two datasets may indicate that expression of at least some of these genes may be regulated by the addition of PUFAs both in vitro and in vivo. Interestingly, some genes showed an inverse regulation in comparison with the data of Kuloyo and co-workers [69]. These were *ALS1*, *CUP9*, *ECM17*, *TYE7* and *YWP1* which were up-regulated in vitro by 20:4n-6, but down-regulated by EPA in vivo. *PST1* was down-regulated by 20:4n-6 in vitro, but up-regulated by EPA in vivo. All of these data indicate that *C.* *albicans* in EPA-supplemented nematodes has a unique expression profile for filamentation-associated genes, that may be partially driven by PUFA supplementation. However, the specific roles of these genes during infection in the presence of EPA need further study.

It is known that pathogenesis can also be influenced by the host immune response. In mammals, EPA is well known for its immunomodulatory effects via production of anti-inflammatory eicosanoids [42, 84, 85]. In *C.* *elegans,* gamma-linolenic acid (18:3n-6) and stearidonic acid (18:4n-3) are essential for the p38 MAP kinase pathway basal activity and immunity against *P.* *aeruginosa* [53] and *fat-6* involved in the synthesis of 18:1n-9, is essential for the induction of innate immune genes [54]. Although EPA is not involved in this, we examined the effect of EPA supplementation on expression of immune response genes during infection with *C.* *albicans*. Several genes involved in immune response, including *cyp-37B1*, *daf-16*, *fipr-22*, *ilys-2*, *lys-5*, *lys-6*, *spp-12,* *fat-3* and *fat-6* were up-regulated (Table 2). Interestingly, *cyp-37B1* is one of 12 core immune response gene of *C.* *elegans* [49] and up-regulation of *daf-16* by PUFAs was also observed previously [64]. Moreover, the observed up-regulation of *fat-3*, which produces 18:3n-6 and 18:4n-3, as well as *fat-6*, indicate the indirect involvement of EPA on lipid mediated immunity. We also observed that *nhr-49*, which functions to regulate lipid metabolism and stress response [43–45], was up-regulated (Table 2). Interestingly, another n-3 fatty acid, 18:3n-3, (which is increased in the nematodes supplemented with EPA) activates NHR-49, increasing expression of genes involved in beta-oxidation either directly or through the production of oxidised metabolites. This has a positive effect on the lifespan of *C.* *elegans* [56]. These results suggest that supplementation with EPA primes the immune response of *C.* *elegans*.

In this study, it is clear that EPA had an effect on the survival of *C.* *albicans*-infected nematodes (increasing the time needed to kill 50% of the infected nematodes) and on hyphal formation via its metabolite, 17,18-EpETE. Yet, further studies on transcriptome profiling, usage of collections of *C.* *albicans* over-expression or knock-out mutants, and target purification will be required to specifically work out the targets of EPA and 17,18-EpETE. Identification of such targets might lead to finding additional inhibitors of fungal morphogenesis with broader applications. Moreover, non-toxic small molecules, such as FAs, that are able to inhibit yeast to hyphal conversion and hyphal growth of *C.* *albicans* could lead to the understanding of pathogenic fungal morphogenesis and may serve as templates for the novel antifungal agents’ development given the rapid emergence of drug-resistant microorganisms.

## Supplementary Information

Below is the link to the electronic supplementary material.

Supplementary file1 (DOCX 44 KB)

Supplementary file2 (XLSX 11 KB)

Supplementary file3 (XLSX 11 KB)

Supplementary file4 (XLSX 11 KB)

Supplementary file5 (XLSX 14 KB)

Supplementary file6 (XLSX 11 KB)

Supplementary file7 (DOCX 32 KB)

## Data Availability

All data generated during this study are included in this article and supplementary data.
