# Supplementary material for: Eicosapentaenoic acid influences the pathogenesis of Candida albicans in Caenorhabditis elegans via inhibition of hyphal formation and stimulation of the host immune response
Source: Med Microbiol Immunol. 2023 Sep 6;212(5):349–68. doi: 10.1007/s00430-023-00777-6 (PMC10501937; doi:10.1007/s00430-023-00777-6)
Supplement: Supplementary file 1 — Supplementary file1 (DOCX 44 KB) [file 430_2023_777_MOESM1_ESM.docx]

**Supplementary Table S1.** *Caenorhabditis elegans* and *Candida albicans* genes used in this study

| ***Caenorhabditis elegans*** | | |
| --- | --- | --- |
| **Reference genes** | | |
| **Probe Name** | **Process/Description** | **References** |
| *rps-2* | Predicted structural constituent of ribosome | 1,[2](http://www.wormbase.org) |
| *rps-4* | Predicted structural constituent of ribosome | 1,[2](http://www.wormbase.org) |
| *rps-23* | Predicted structural constituent of ribosome | 1,[2](http://www.wormbase.org) |
| **Lipid metabolism** | | |
| **Probe Name** | **Process/Description** | **References** |
| *cyp-29A2* | Cytochrome P450, involved in lipid storage | 2,3 |
| *cyp-29A3* | Cytochrome P450, involved in eicosapentaenoic acid (EPA) metabolism | 2,4 |
| *cyp-32A1* | Cytochrome P450, involved in reproduction and possibly eicosanoid production | 2,5 |
| *cyp-33C1* | Cytochrome P450 | 2 |
| *cyp-33E1* | Cytochrome P450, orthologue of *cyp-33E2*, possibly involved in long-chain fatty acid metabolic process | 2 |
| *cyp-33E2* | Cytochrome P450, involved in long chain fatty acid metabolic process and regulation of pharyngeal pumping | 2 |
| *cyp-37A1* | Cytochrome P450, expressed in intestine, involved in lipid storage and life span | 2,3 |
| *cyp-42A1* | Cytochrome P450, involved in lipid metabolism and protection against glucotoxicity | 2,6 |
| *elo-1* | Predicted fatty acid elongase, involved in unsaturated fatty acid biosynthetic process | 2 |
| *elo-2* | Predicted fatty acid elongase, involved in several processes, including determination of adult lifespan, lipid metabolic process and reproduction | 2 |
| *elo-3* | Predicted fatty acid elongase | 2 |
| *elo-4* | Predicted fatty acid elongase | 2 |
| *elo-5* | Predicted fatty acid elongase, expressed in amphid neurons and intestine | 2 |
| *elo-6* | Predicted fatty acid elongase, expressed in amphid neurons, intestine, nerve ring and vulva | 2 |
| *elo-7* | Predicted fatty acid elongase | 2 |
| *elo-8* | Predicted fatty acid elongase | 2 |
| *elo-9* | Predicted fatty acid elongase | 2 |
| *emb-8* | NADPH-cytochrome P450 reductase, involved in eggshell formation and lipid biosynthetic process | 2 |
| *fat-1* | Omega-3 fatty acid desaturase involved in positive regulation of locomotion and unsaturated fatty acid biosynthetic process | 2 |
| *fat-2* | Delta 12-fatty acid dehydrogenase and stearoyl-CoA 9-desaturase involved in fatty acid biosynthetic process and innate immune response | 2 |
| *fat-3* | Stearoyl-CoA 9-desaturase | 2 |
| *fat-4* | Stearoyl-CoA 9-desaturase involved in unsaturated fatty acid biosynthetic process | 2 |
| *fat-5* | Predicted to enable iron ion binding activity and stearoyl-CoA 9-desaturase activity, involved in long-chain fatty acid biosynthetic process | 2 |
| *fat-6* | Stearoyl-CoA 9-desaturase activity, involved in innate immune response, long-chain fatty acid biosynthetic process and multicellular organism development | 2 |
| *fat-7* | Stearoyl-CoA 9-desaturase activity, involved in fatty acid biosynthetic process | 2 |
| *mboa-7* | Enables O-acyltransferase activity. Is involved in nematode larval development, oviposition and phosphatidylinositol biosynthetic process. | 2 |
| *nhr-49* | Involved in determination of adult lifespan, positive regulation of transcription from RNA polymerase II promoter in response to stress, regulation of fatty acid metabolic process and regulates immuno-metabolic response to bacterial infection | 2,7 |
| **Immune response** | | |
| **Probe Name** | **Process/Description** | **References** |
| *abf-2* | Involved in defence response to Gram-negative and Gram-positive bacteria | 2 |
| *abf-3* | Involved in innate immune response to Gram-negative bacteria | 2 |
| *atf-7* | Involved in several processes, including defence response to Gram-negative bacterium, regulation of innate immune response and regulation of transcription by RNA polymerase II | 2 |
| *cht-1* | Chitinase involved in response to fungus | 2 |
| *clec-60* | C-type lectin involved in defence response to Gram-positive bacteria, expressed in intestine | 2 |
| *clec-67* | C-type lectin involved in PERK-mediated unfolded protein response. Is expressed in intestine | 2 |
| *cnc-2* | Caenacin involved in defence response to Gram-negative bacterium, defence response to fungus and innate immune response, expressed in hypodermis | 2 |
| *cnc-4* | Caenacin involved in defence response to fungus and innate immune response | 2 |
| *cnc-5* | Caenacin involved in defence response to fungus and innate immune response | 2 |
| *cnc-6* | Caenacin involved in defence response to fungus and innate immune response | 2 |
| *col-179* | Predicted extracellular matrix structural constituent, involved in defence response to Gram-negative bacterium and innate immune response | 2 |
| *cyp-14A2* | Cytochrome P450, expressed in intestine, possibly involved in stress response and detoxification | 2,8 |
| *cyp-37B1* | Cytochrome P450, involved in defence response to Gram-positive bacteria | 2 |
| *daf-16* | Involved in several processes, including defence response to other organisms | 2 |
| *fipr-22* | Fungus induced peptide enriched in GABAergic neurons, excretory cell and hypodermis | 2 |
| *ilys-2* | Lysozyme involved in defence response to Gram-positive bacterium | 2 |
| *ilys-5* | Lysozyme | 2 |
| *lys-2* | Lysozyme involved in defence response to Gram-negative and Gram-positive bacteria | 2 |
| *lys-4* | Lysozyme involved in defence response to Gram-positive bacterium | 2 |
| *lys-5* | Lysozyme involved in defence response to Gram-positive bacteria | 2 |
| *lys-6* | Lysozyme | 2 |
| *lys-7* | Lysozyme expressed in head neurons intestine and rectal gland cell | 2 |
| *spp-1* | Caenopore involved in defence response to other organism and pore formation in membrane of another organism | 2 |
| *spp-2* | Caenopore involved in defence response to Gram-positive bacteria | 2 |
| *spp-3* | Caenopore involved in defence response to Gram-negative and Gram-positive bacteria | 2 |
| *spp-4* | Caenopore enriched in hypodermis and intestine | 2 |
| *spp-8* | Caenopore enriched in outer labial lateral neurons, polymodal nociceptive neurons and intestine | 2 |
| *spp-12* | Caenopore involved in defence response to Gram-positive bacteria | 2 |
| *spp-14* | Caenopore involved in immune response | 2 |
| *spp-23* | Caenopore enriched in dorsal A neuron, ventral A neuron, intestine and pharyngeal muscle cell | 2 |
| ***Candida albicans*** | | |
| **Reference genes** | | |
| **Probe Name** | **Process/Description** | **References** |
| *ACT1* | Actin | [9](http://www.candidagenome.org),10 |
| *LSC2* | Putative succinate-CoA ligase beta subunit | [9](http://www.candidagenome.org),10 |
| *THD3* | NAD-linked glyceraldehyde-3-phosphate dehydrogenase | [9](http://www.candidagenome.org),10 |
| **Filamentation** | | |
| **Probe Name** | **Process/Description** | **References** |
| *ACE2* (orf19.6124) | Transcription factor that regulates morphogenesis, mutant is hyperfilamentous | 9 |
| *ADA2* (orf19.2331) | Transcriptional coactivator, role in cell wall integrity. Mutation prevents hyphal formation in *Caenorhabditis elegans* | 9 |
| *ADR1* (orf19.2752) | Transcription factor, transposon mutation affects filamentous growth | 9 |
| *AFT*2 (orf19.2272) | Putative transcription factor with role in hyphal growth | 9 |
| *AHR1* (orf19.7381) | Transcription factor, involved in regulation of adhesion genes. Mutation affects hyphal growth | 9 |
| *ALS1* (orf19.5741) | Cell-surface adhesin with role in biofilm formation and hyphal formation. Arachidonic acid (AA) responsive gene | [9](http://www.candidagenome.org),11 |
| *ARG81* (orf19.4766) | Transcription factor, required for ornithine utilisation. Mutation may increase filamentation in certain strains. | 9 |
| *ALS3* (orf19.1816) | Cell wall adhesion and hyphal-associated invasin, mutation causes defect in biofilm formation | 9 |
| *ASG1*  (orf19.166) | Transcription factor, mutation causes decreased filamentation in certain strains | 9 |
| *ASH1* (orf19.5343) | Transcription factor required for filamentous growth on solid media | 9 |
| *BCR1*  (orf19.723) | Transcription factor, regulates biofilm formation and involved in hyphal growth | 9 |
| *BRE1*  (orf19.976) | Putative E3 ubiquitin ligase, involved in ubiquitination of histone H2B during hyphal development, transposon mutation affects filamentous growth | 9 |
| *BRG1* (orf19.4056) | Transcription factor, transposon mutation affects filamentation | 9 |
| *CAS5* (orf19.4670) | Transcription factor, mutants have hyphal defect in *C. elegans* infection. AA responsive gene | [9](http://www.candidagenome.org),11 |
| *CPH1* (orf19.4433) | Transcription factor involved in filamentation on solid media | 9 |
| *CPH2* (orf19.1187) | Transcription factor, promotes hyphal growth | 9 |
| *CRZ1* (orf19.7359) | Calcineurin-regulated transcription factor, mutation decreases hyphal formation in certain strains. AA responsive gene | [9](http://www.candidagenome.org),11 |
| *CSR1* (orf19.3794) | Transcription factor, mutation affects filamentous growth | 9 |
| *CTA4* (orf19.7374) | Transcription factor, involved in mating. Mutants may Mutations cause decreased filamentation. AA responsive gene | [9](http://www.candidagenome.org),11 |
| *CTA8* (*HSF1*) (orf19.4775) | Essential transcription factor, mutation may cause increased hyphal growth in certain strains | 9 |
| *CUP9* (orf19.6514) | Transcription factor, mutants have increased filamentous growth. AA responsive gene | [9](http://www.candidagenome.org),11 |
| *CWT1* (orf19.5849) | Transcription factor, mutation causes decreased filamentous growth in certain strains | 9 |
| *CZF1* (orf19.3127) | Hyphal growth regulator. AA responsive gene | [9](http://www.candidagenome.org),11 |
| *EFG1*  (orf19.610) | Transcription factor, required for hyphal growth | 9 |
| *EFH1* (orf19.5498) | Transcription factor, regulates filamentous growth | 9 |
| *ERG11* (orf19.922) | Cytochrome P450 family, role in ergosterol biosynthesis and hyphal formation. AA responsive gene | [9](http://www.candidagenome.org),11 |
| *FCR1* (orf19.6817) | Transcription factor, transposon mutation enhances filamentation. Polyunsaturated fatty acid AA responsive gene | [9](http://www.candidagenome.org),11 |
| *FGR13* (orf19.1006) | Transposon mutation affects filamentous growth | 9 |
| *FGR17* (orf19.5729) | Putative transcription factor, transposon mutation affects filamentous growth | 9 |
| *FGR27* (orf19.6680) | Transcription factor, transposon mutation affects filamentous growth | 9 |
| *FKH2* (orf19.5389) | Transcription factor, mutant lacks true hyphae, is constitutively pseudohyphal. | 9 |
| *FLO8* (orf19.1093) | Transcription factor required for hyphal formation, regulates hyphal gene expression | 9 |
| *GPR1* (orf19.1944) | Plasma membrane G-protein-coupled receptor required for wild type hyphal growth | 9 |
| *GRF10* (orf19.4000) | Putative transcription factor, involved in control of filamentous growth | 9 |
| *HAP5* (orf19.1973) | Transcription factor with roles in filamentous growth | 9 |
| *HMS1*  (orf19.921) | Transcript factor required for morphogenesis induced by elevated temperature | [9](http://www.candidagenome.org) |
| *HOT1* (orf19.3328) | Required for inhibition of filamentous growth by farnesoic acid, filament induced | 9 |
| *HWP1* (orf19.13.21) | Hyphal cell wall protein | 9 |
| *MED7*  (orf19.232) | Subunit of the RNA polymerase II mediator complex. Mutations may affect filamentation in certain strains | 9 |
| *MSS11* (orf19.6309) | Transcription factor required for hyphal growth | 9 |
| *NDT80* (orf19.2119) | Activator of *CDR1* induction by antifungal drugs, required for Spider biofilm formation and hyphal formation. AA responsive gene | [9](http://www.candidagenome.org),11 |
| *NGS1* (orf19.7516) | Acts as N-acetylglucosamine (GlcNAc) sensor required for GlcNAc-induced filamentation | 9 |
| *NOT3* (orf19.2012) | Transcriptional regulator, mutant colonies exhibit slightly decreased filamentation ratio | 9 |
| *NOT5* (orf19.5107) | Member of the transcription regulatory CCR4-NOT complex, required for hyphal growth | 9 |
| *NRG1* (orf19.7150) | Transcription factor/repressor, regulates hyphal gene induction. AA responsive gene | [9](http://www.candidagenome.org),11 |
| *OFI1*  (orf19.4972) | Putative transcription factor involved in regulation of filamentous growth | 9 |
| *OPI1*  (orf19.1543) | Transcription factor, involved in regulation of filamentous growth | 9 |
| *PGA13* (orf19.6420) | GPI-anchored cell wall protein involved in cell wall synthesis. Mutation may cause delayed filamentation in certain strains. AA responsive gene | [9](http://www.candidagenome.org),11 |
| *PHO4* (orf19.1253) | Transcription factor, required for phosphate acquisition and stress resistance. Mutations may cause increased filamentous growth in certain strains | 9 |
| *PPR1* (orf19.3986) | Transcription factor, mutants have decreased hyphal growth in certain strains | 9 |
| *RBF1* (orf19.5558) | Transcription factor, mutation causes accelerated induction of filamentous growth | 9 |
| *RCA1* (orf19.6102) | Protein involved in regulation of carbonic anhydrases. mutation affects filamentous growth | 9 |
| *RFG1* (orf19.2823) | Transcriptional repressor of filamentous growth and hyphal genes. Possible AA responsive gene | [9](http://www.candidagenome.org),11 |
| *RFX2* (orf19.4590) | Transcriptional repressor, regulator of filamentation. AA responsive gene | [9](http://www.candidagenome.org),11 |
| *RIM101* (orf19.7247) | Transcription factor, required for alkaline-induced hyphal growth. Possible AA responsive gene | [9](http://www.candidagenome.org),11 |
| *RLM1* (orf19.4662) | Transcription factor, mutation causes decreased hyphal growth on lactate | 9 |
| *ROB1* (orf19.4998) | Transcription factor, mutant displays abnormal colony morphology (no peripheral hyphae) and invasive growth. Possible AA responsive gene | [9](http://www.candidagenome.org),11 |
| *RON1*  (orf19.513) | Required for GlcNAc-induced hyphal growth | 9 |
| *RTG3* (orf19.2315) | Transcription factor, mutation causes decreased filamentous growth in certain strains | 9 |
| *SAP6* (orf19.5542) | Expressed during hyphal growth, involved in biofilm formation, AA responsive gene | [9](http://www.candidagenome.org),11 |
| *SEF1* (orf19.3753) | Transcription factor, regulates iron uptake. Mutation may decrease filamentation in certain strains. | 9 |
| *SET3* (orf19.7221) | NAD-dependent histone deacetylase, mutations affect filamentous growth. AA responsive gene | [9](http://www.candidagenome.org),11 |
| *SFL1*  (orf19.454) | Transcription factor involved in negative regulation of morphogenesis. AA responsive gene | [9](http://www.candidagenome.org),11 |
| *SFL2* (orf19.3969) | Transcription factor involved in regulation of morphogenesis, required for filamentous growth | 9 |
| *SIN3*  (orf19.6011) | Transposon mutation affects filamentous growth | 9 |
| *SKN7*  (orf19.971) | Predicted response regulator protein. Mutation causes absence of hyphal formation in certain strains | 9 |
| *SKO1* (orf19.1032) | Transcription factor, represses the yeast-to-hypha transition | 9 |
| *SMI1* (orf19.5058) | Cell wall biosynthesis protein, Cyr1-induced in hyphal cells | 9 |
| *SNF4* (orf19.5768) | Putative subunit of the AMP-activated Snf1p kinase, transposon mutation affects filamentation | 9 |
| *SNF5* (orf19.5871) | Part of SWI/SNF complex which is essential for hyphal growth | 9 |
| *SNF6*  (orf19.831) | Part of SWI/SNF complex which is essential for hyphal growth | 9 |
| *SNQ2* (orf19.5759) | Transporter, transposon mutation affects filamentation. AA responsive gene | [9](http://www.candidagenome.org),11 |
| *SPT3* (orf19.7622) | Homozygous null mutant is hyperfilamentous | 9 |
| *SPT6* (orf19.7136) | Putative transcription elongation factor, transposon mutation affects filamentous growth | 9 |
| *SPT20* (orf19.422) | Putative transcription factor, mutants have decreased hyphal and invasive growth | 9 |
| *SSN6* (orf19.6798) | Hyphal growth regulator, repressed during hyphal growth | 9 |
| *STD1* (orf19.6173) | Putative transcription factor, mutation causes increased filamentation | 9 |
| *STP2* (orf19.4961) | Amino-acid-regulated transcription factor. Mutation decreases filamentation | 9 |
| *SWI1* (orf19.5657) | Part of SWI/SNF complex which is essential for hyphal growth | 9 |
| *SWI4* (orf19.4545) | Putative component of the SBF transcription complex involved in G1/S cell-cycle progression, mutants have reduced peripheral filamentation | 9 |
| *TAC1* (orf19.3188) | Zn(2)-Cys(6) transcriptional activator of drug-responsive genes, mutation causes decreased hyphal formation in certain strains. AA responsive gene | [9](http://www.candidagenome.org),11 |
| *TCC1* (orf19.6734) | Putative transcription factor, regulation of filamentation and expression of hypha-specific genes | 9 |
| *TEA1* (orf19.6985) | Putative transcription factor, mutation may cause increased hyphal growth in certain strains | 9 |
| *TEC1* (orf19.5908) | Transcription factor involved in hyphal gene regulation. AA responsive gene | [9](http://www.candidagenome.org),11 |
| *TFG1* (orf19.4585) | Transposon mutation affects filamentous growth | 9 |
| *TUP1* (orf19.6109) | Transcriptional corepressor, represses filamentous growth, role in germ tube induction | 9 |
| *UME6* (orf19.1822) | Transcription factor that regulates filamentous growth | 9 |
| *WOR1* (orf19.4884) | Transcription factor ("master switch") of white-opaque phenotypic switching. Mutants have decreased biofilm formation in certain strains | 9 |
| *YOR1* (orf19.1783) | ABC-type plasma membrane transporter involved in resistance to aureobasidin A. Mutation causes a decrease in invasive growth. AA responsive gene | [9](http://www.candidagenome.org),11 |
| *ZCF3* (orf19.1168) | Transcription factor required for filamentous growth | 9 |
| *ZCF7* (orf19.1685) | Predicted transcription factor, mutation causes decreased colony wrinkling and hyphal formation. | 9 |
| *ZCF11* (orf19.2423) | Transcription factor required for wild-type filamentous growth | 9 |
| *ZCF14* (orf19.2647) | Putative transcription factor, mutants have decreased hyphal growth | 9 |
| *ZCF17* (orf19.3305) | Putative transcription factor, mutants have increased hyphal growth, but decreased invasive growth | 9 |
| *ZCF18* (orf19.3405) | Putative transcription factor, mutants have increased hyphal growth | 9 |
| *ZCF29* (orf19.5133) | Transcription factor, mutants have defects in filamentous growth | 9 |
| *ZCF32* (orf19.5940) | Transcription factor involved in regulation of biofilm formation | 9 |
| *ZNC1* (orf19.3187) | Transcription factor required for adherence and represses hyphal cell formation | 9 |
| **Other processes** | | |
| **Probe Name** | **Process/Description** | **References** |
| *CDR1* (orf19.6000) | Multidrug transporter of ABC superfamily, transport phospholipids. AA responsive gene | 9,11 |
| *CDR2* (orf19.5958) | Multidrug transporter, ATP-binding cassette (ABC) superfamily, transports phospholipids. AA responsive gene | [9](http://www.candidagenome.org),11 |
| *DAL8* (orf19.5859) | Putative allantoate permease, fungal-specific, AA responsive gene | [9](http://www.candidagenome.org),11 |
| *EHT1* (orf19.3040) | Putative acyl-coenzymeA: ethanol O-acyltransferase. AA responsive gene | [9](http://www.candidagenome.org),11 |
| *ECM17* (orf19.4099) | Putative sulfite reductase beta subunit, role in cell wall biogenesis. AA responsive gene | [9](http://www.candidagenome.org),11 |
| *FAH2*  orf19.2184 | Putative fumarylacetoacetate hydrolase | 9 |
| *FLU1* (orf19.6577) | Multidrug efflux pump of the plasma membrane AA responsive gene | [9](http://www.candidagenome.org),11 |
| *HRD3*  orf19.1191 | Ortholog(s) have ubiquitin-protein transferase activity and role in negative regulation of protein autoubiquitination | 9 |
| *HSP12* (orf19.3160) | Decreased expression in hyphae. AA responsive gene | [9](http://www.candidagenome.org),11 |
| *IPT1*  (orf19.4769) | Inositol phosphoryl transferase, catalyzes the synthesis of the most abundant sphingolipid. AA responsive gene | [9](http://www.candidagenome.org),11 |
| *MET4* (orf19.5312) | Putative transcription coactivator, predicted role in sulphur amino acid metabolism. AA responsive gene | [9](http://www.candidagenome.org),11 |
| *MDR1* (orf19.5604) | Plasma membrane MDR/MFS multidrug efflux pump. AA responsive gene | [9](http://www.candidagenome.org),11 |
| *PDR16* (orf19.1027) | Phosphatidylinositol transfer protein. AA responsive gene | [9](http://www.candidagenome.org),11 |
| *PST1* (orf19.2241) | Hyphal-induced. AA responsive gene | [9](http://www.candidagenome.org),11 |
| *RGT1* (orf19.4722) | Transcription factor, transcriptional repressor involved in the regulation of glucose transporter genes | 9 |
| *RTA3*  (orf19.23) | 7-transmembrane receptor protein involved in regulation of asymmetric lipid distribution in plasma membrane. AA responsive gene | [9](http://www.candidagenome.org),11 |
| *SUL2* (orf19.2738) | Putative sulfate transporter. AA responsive gene | [9](http://www.candidagenome.org),11 |
| *SUT1* (orf19.4342) | Transcription factor involved in sterol uptake. AA responsive gene | [9](http://www.candidagenome.org),11 |
| *TYE7* (orf19.4941) | Transcription factor, control of glycolysis. AA responsive gene | [9](http://www.candidagenome.org),11 |
| *UGA3* (orf19.7570) | Transcription factor, required for utilization of gamma-aminobutyrate | 9 |
| *UPC2*  (orf19.391) | Transcription factor, regulates ergosterol biosynthesis | 9 |
| *YWP1* (orf19.3618) | Secreted yeast wall protein, involved in adhesion and biofilm formation. AA responsive gene | [9](http://www.candidagenome.org),11 |

1. Tao J, Hao Y, Li X, Yin H, Nie X, Zhang J, Xu B, Chen Q, Li B. 2020. Systematic identification of housekeeping genes possibly used as references in *Caenorhabditis elegans* by large-scale data integration. *Cells* 9:786.

2. WormBase. https://wormbase.org/#012-34-5

3. Zhang Y, Zou X, Ding Y, Wang H, Wu X, Liang B. 2013. Comparative genomics and functional study of lipid metabolic genes in *Caenorhabditis elegans. BMC Genomics* 14:164.

4. Liberman N, O'Brown ZK, Earl AS, Boulias K, Gerashchenko MV, Wang SY, Fritsche C, Fady PE, Dong A, Gladyshev VN, Greer EL. 2020. N6-adenosine methylation of ribosomal RNA affects lipid oxidation and stress resistance. *Sci Adv* 6(17):eaaz4370.

5. Benenati G, Penkov S, Müller-Reichert T, Entchev EV, Kurzchalia TV. 2009. Two cytochrome P450s in *Caenorhabditis elegans* are essential for the organization of eggshell, correct execution of meiosis and the polarization of embryo. *Mech Dev* 126(5-6):382–393.

6. Jung Y, Kwon S, Ham S, Lee D, Park HH, Yamaoka Y, Jeong DE, Artan M, Altintas O, Park S. et al. 2020. *Caenorhabditis elegans* Lipin 1 moderates the lifespan-shortening effects of dietary glucose by maintaining ω-6 polyunsaturated fatty acids. *Aging cell* 19(6):e13150.

7. Van Gilst MR, Hadjivassiliou H, Jolly A, Yamamoto KR. 2005. Nuclear hormone receptor NHR-49 controls fat consumption and fatty acid composition in *C. elegans*. *PLoS Biol* 3:e53.

8. Thomas JH. 2007. Rapid birth-death evolution specific to xenobiotic cytochrome P450 genes in vertebrates. *PLoS Genet* 3:e67.

9. Candida Genome Database. http://72/

10. Nailis H, Coenye T, Van Nieuwerburgh F, Deforce D, Nelis HJ. 2006. Development and evaluation of different normalization strategies for gene expression studies in *Candida albicans* biofilms by real-time PCR. *BMC Mol Biol* 7:25.

11. Kuloyo O, Fourie R, Cason E, Albertyn J, Pohl CH. 2020. Transcriptome analyses of *Candida albicans* biofilms, exposed to arachidonic acid and fluconazole, indicates potential drug targets. *G3* 10:3099-3108.
