# Supplementary material for: Eicosapentaenoic acid influences the pathogenesis of Candida albicans in Caenorhabditis elegans via inhibition of hyphal formation and stimulation of the host immune response
Source: Med Microbiol Immunol. 2023 Sep 6;212(5):349–68. doi: 10.1007/s00430-023-00777-6 (PMC10501937; doi:10.1007/s00430-023-00777-6)
Supplement: Supplementary file 7 — Supplementary file7 (DOCX 32 KB) [file 430_2023_777_MOESM7_ESM.docx]

**Fig S1** Effect of 17,18-EpETE on germ tube formation of *Candida albicans* using a crystal violet germ tube assay*.* Values represents the mean of three independent experiments and error bars represent the standard deviations. Asterisk (*) indicate *P* < 0.05 compared to 17,18-EpETE unsupplemented media.
